# Leaky Gut Plays a Critical Role in the Pathophysiology of Autism in Mice by Activating the Lipopolysaccharide-Mediated Toll-Like Receptor 4–Myeloid Differentiation Factor 88–Nuclear Factor Kappa B Signaling Pathway

**DOI:** 10.1007/s12264-022-00993-9

**Published:** 2022-12-18

**Authors:** Fang Li, Haoran Ke, Siqi Wang, Wei Mao, Cexiong Fu, Xi Chen, Qingqing Fu, Xiaori Qin, Yonghua Huang, Bidan Li, Shibing Li, Jingying Xing, Minhui Wang, Wenlin Deng

**Affiliations:** 1 Department of Gastroenterology, Gastroenterology Endoscopy Center, Hainan General Hospital, Hainan Affiliated Hospital of Hainan Medical University, Haikou, 570311 China; 2 Hepatology Unit, Department of Infectious Diseases, Nanfang Hospital, Southern Medical University, Guangzhou, 510515 China; 3 Department of Gastroenterology, First Affiliated Hospital of Guangzhou Medical University, Guangzhou Medical University, Guangzhou, 510120 China; 4 Department of Hepatopancreatobiliary Surgery, Hainan General Hospital, Hainan Affiliated Hospital of Hainan Medical University, Haikou, 570311 China; 5 Otolaryngology-Head and Neck Surgery, Hospital of Hainan Province, Department of Otorhinolaryngology, Head and Neck Surgery, Hainan General Hospital, Hainan Affiliated Hospital of Hainan Medical University, Haikou, 570311 China; 6 Department of Radiology, Hainan General Hospital, Hainan Affiliated Hospital of Hainan Medical University, Haikou, 570311 China; 7 Department of Pediatric Surgery, Hainan General Hospital, Hainan Affiliated Hospital of Hainan Medical University, Haikou, 570311 China; 8 Department of Gastroenterology, Hainan General Hospital, Hainan Affiliated Hospital of Hainan Medical University, Haikou, 570311 China; 9 Department of Nephrology, Department of Nephrology, Hainan Affiliated Hospital of Hainan Medical College, Hainan General Hospital, Haikou, 570311 China; 10 Department of Pediatrics, The Sixth Affiliated Hospital, Sun Yat-sen University, Guangzhou, 510655 China

**Keywords:** Autism, Gut, Lipopolysaccharide, Toll-like receptor 4, Metformin

## Abstract

Increased intestinal barrier permeability, leaky gut, has been reported in patients with autism. However, its contribution to the development of autism has not been determined. We selected dextran sulfate sodium (DSS) to disrupt and metformin to repair the intestinal barrier in BTBR T^+^tf/J autistic mice to test this hypothesis. DSS treatment resulted in a decreased affinity for social proximity; however, autistic behaviors in mice were improved after the administration of metformin. We found an increased affinity for social proximity/social memory and decreased repetitive and anxiety-related behaviors. The concentration of lipopolysaccharides in blood decreased after the administration of metformin. The expression levels of the key molecules in the toll-like receptor 4 (TLR4)–myeloid differentiation factor 88 (MyD88)–nuclear factor kappa B (NF-κB) pathway and their downstream inflammatory cytokines in the cerebral cortex were both repressed. Thus, “leaky gut” could be a trigger for the development of autism *via* activation of the lipopolysaccharide-mediated TLR4–MyD88–NF-κB pathway.

**Supplementary Information:**

The online version contains supplementary material available at 10.1007/s12264-022-00993-9.

## Introduction

Autism falls under autism spectrum disorder and is characterized by repetitive behaviors and difficulties with social communication [1] that can affect multiple systems in the body, including the nervous system, immune system, and gastrointestinal tract [2]. The prevalence of autism has dramatically increased during the past few decades [3], but its causes are largely unknown. There is currently no effective way to treat the core symptoms of autism.

In addition to the aforementioned features, individuals with autism spectrum disorder (ASD) often suffer from gastrointestinal problems [4]. Studies over the past decade have demonstrated that autistic children experience gastrointestinal symptoms 4.4 times more often than neurotypical children; this mainly includes constipation, diarrhea, and abdominal pain [5]. A large meta-analysis comparing autism cases with healthy controls from 1980 to 2012 and a multicenter study with >14,000 ASD individuals both reported this phenotype [6]. Furthermore, GI symptoms tend to be strongly correlated with the severity of autism and with increased irritability, anxiety, and social withdrawal [7]. The frequency of gastrointestinal problems was associated with greater social withdrawal, stereotypy, irritability, and hyperactivity in an examination of 960 children from the Childhood Autism Risks from Genetics and Environment (CHARGE) study [8]. Thus, scientists have reached a consensus that gastrointestinal problems may potentiate autistic behavioral issues [9–11]. This evidence suggests that gut dysfunction may be a major promotor of the behavioral symptoms of autism. This conclusion is consistent with the concept of the "gut–brain" axis proposed in recent years [12–14].

The ‘gut–brain’ axis describes the bidirectional physiological connection that helps information exchange between the gut and the brain [12]. For instance, studies have reported that lipopolysaccharides (LPS), metabolites of gram-negative bacteria, can pass through the damaged intestinal barrier into the brain and induce a pro-inflammatory environment that can affect brain function [12, 15]. The gastrointestinal mucosa forms a barrier between the body and the lumenal environment *via* intestinal epithelial cells connected through tight junctions (TJs) and a mucus layer, which separates trillions of microorganisms from the body. The exclusionary properties of the gastrointestinal mucosa are referred to as the gastrointestinal barrier, which is considered to be the core link between the gut and the brain in the gut–brain axis [16]. Dysbiosis of microbiota has not only been reported in gut-related diseases such as inflammatory bowel disease and irritable bowel syndrome, but also in neurological and mental disorders, like stress, depression, Alzheimer’s disease, Parkinson’s disease, and ASD. In addition to dysbiosis, the integrity of intestinal barrier composition is also important to health. Dysfunction of the intestinal barrier has been reported in patients with autism and even in first-degree relatives of affected individuals; a high percentage of abnormal intestinal permeability values, measured by calculating the lactulose/mannitol ratio, has been found in patients with autism and their relatives when compared with healthy controls [17]. D’ Eufemia *et al.* also found damage to TJs of the gut in autistic children and indicated that in some patients with infantile autism, damage to these junctions occurs in the absence of gastrointestinal disorders [18]. However, another recent study showed no abnormal intestinal permeability in autistic children compared with healthy siblings and unrelated controls [19]. Increased intestinal permeability has also been found in some close non-autistic relatives of autistic individuals, suggesting that intestinal integrity is not a consequence of ASD [20]. Thus, it is unclear whether the disruption of the intestinal barrier is a cause or a consequence of autism.

Our results confirmed that increased intestinal barrier permeability, referred to as ‘leaky gut’, does occur in BTBR T^+^tf/J mice that have been used as an animal model of autism [21]. Behavioral manifestations of these autistic mice were significantly relieved after the administration of metformin to repair the intestinal barrier. These results showed that the ‘leaky gut’ may indeed contribute to the development of autism. Moreover, we elucidated the potential mechanism by which gut-originated LPS may pass through the damaged intestinal barrier into the brain to activate the TLR4–mediated myeloid differentiation primary response protein 88–dependent nuclear factor-kappa B (TLR4/MyD88/NF-κB) pathway, resulting in a pro-inflammatory micro-environment, which seems to be an important contributor of autism. The down-regulated expression levels of the key molecules and inflammatory cytokines of the TLR4 signaling pathway in the cerebral cortex, after the intestinal barrier was repaired, support our point.

Few studies have been designed to determine the role of abnormal gut permeability in the neuropsychiatric manifestations of ASD. In this study, we aimed to elucidate the effect of a ‘leaky gut’ on the various autistic behavioral phenotypes, using BTBR mice (an autism model). In addition, we sought to further understand the underlying mechanisms that increase the permeability of the intestinal barrier contributing to autism.

## Materials and Methods

### Animals

All animal experiments were performed according to protocols approved by the Animal Care Committee of Hainan General Hospital and were conducted in accordance with the US National Institutes of Health Guide for the Care and Use of Laboratory Animals (NIH Publication 85–23, revised 2011). Male BTBR T^+^tf/J (BTBR, Jackson Laboratory, Bar Harbor, ME, USA) and C57BL/6 (B6, Southern Medical University, Guangzhou, China) mice, aged 7–8 weeks, and weighing 20–25 g were maintained under controlled light conditions (12:12 light-dark cycle) with a normal chow diet and water provided *ad libitum*.

### Treatment Protocol

**Fig. 1:**
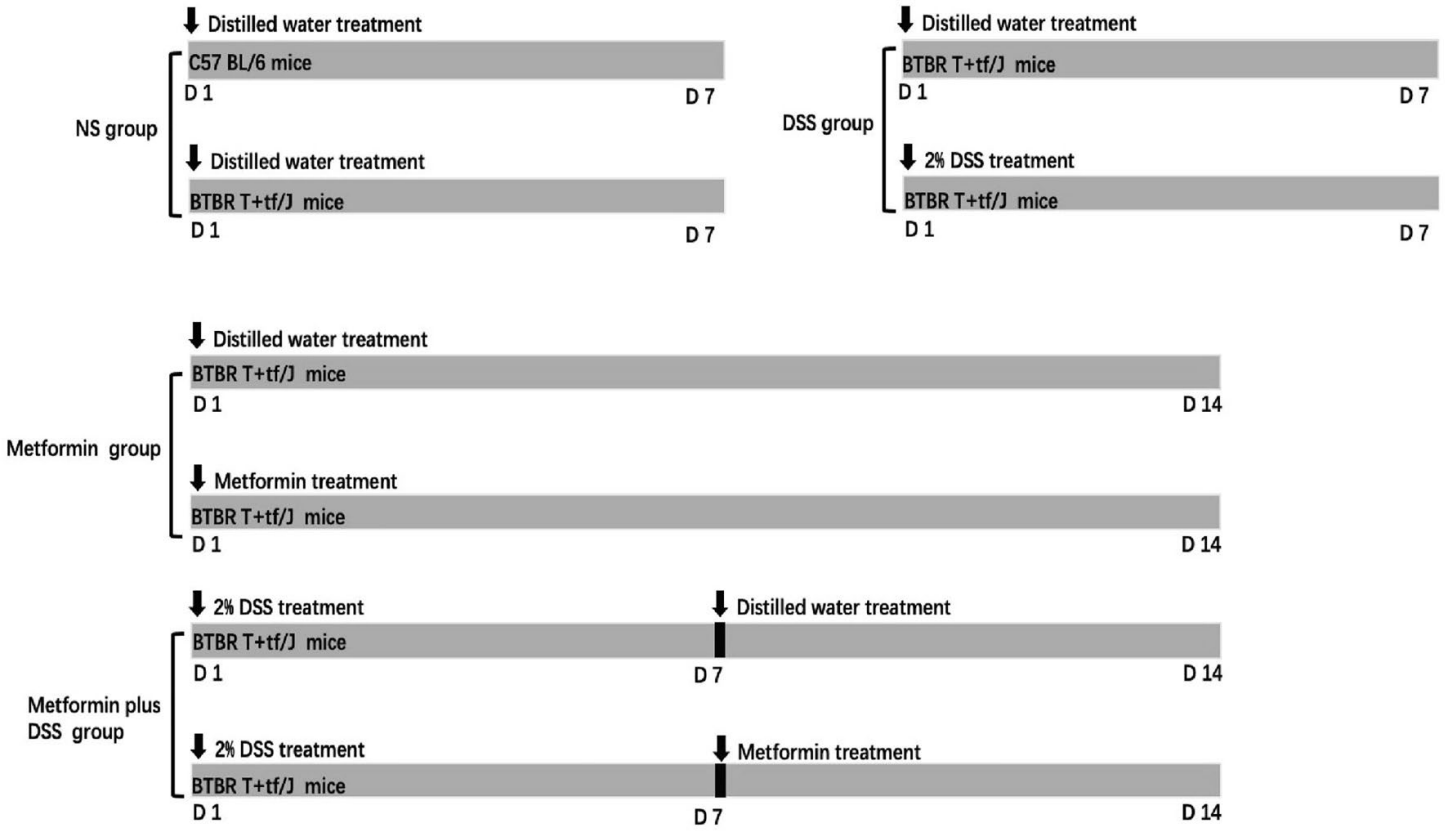
Design of the mouse experiment. The experiments were performed on four groups each composed of 9–13 male C57BL/6J and BTBR T^+^tf/J mice: (1) NS (normal saline) group –BTBR T^+^tf/J and C57 BL/6 mice both received distilled water; (2) DSS group – BTBR T^+^tf/J mice received 2% DSS treatment or distilled water; (3) metformin group – metformin or distilled water was applied to BTBR T^+^tf/J mice by lavage for two weeks; (4) metformin plus DSS group – BTBR T^+^tf/J mice received 2% DSS in the first week followed by metformin treatment or distilled water for the next week. To test our hypothesis, we applied a number of tests on these groups: IHC and RT-PCR, an intestinal barrier permeability test, and behavioral analysis (5 mice per treatment for IHC analysis, 6–8 mice per treatment for FITC dextran testing, 8–13 mice per treatment for behavioral analysis, and 4–8 mice per treatment for RT-PCR analysis.

To explore the effects of intestinal barrier changes on autistic behavior, all animals were divided into four groups as follows (Fig. 1):

(1) NS (normal distilled water) group: BTBR and B6 mice both received distilled water only (BTBR *vs* B6);

(2) DSS group: 2% DSS treatment was administered to BTBR mice daily for one week. Matched, mice of the same strain were administered distilled water daily for the same time (DSS *vs* Distilled water);

(3) Metformin group: metformin was administered to BTBR mice by lavage for two weeks. Matched mice of the same strain were administered distilled water daily for the same time (Met *vs* Distilled water);

(4) Metformin plus DSS group: 2% DSS was administered to BTBR mice in the first week, followed by metformin treatment or distilled water for the next week (DSS+Met *vs* DSS+Distilled water).

During the observation period, the body weight of each mouse was monitored every two days. All the animals were anesthetized and then sacrificed by cervical dislocation by the end of the experiment. The cerebral cortex was carefully removed and immediately frozen in liquid nitrogen for further analysis. Colon tissue was removed and cut into two pieces. One piece was immediately frozen in liquid nitrogen for further analysis. The other piece was fixed in 10% formaldehyde for histopathological study.

### Drugs

Metformin (Sigma-Aldrich, Cat No. 317240) and Dextran Sulfate Sodium Salt (DSS) (Sigma-Aldrich, Cat No. 67578) were from Sigma-Aldrich (St. Louis, Missouri, USA). Metformin was dissolved in H_2_O and stored at −20°C. Mice were dosed with a metformin compound formulation of 400 mg/kg once per day by oral gavage.

### Total RNA Extraction and Real-Time Quantitative Polymerase Chain Reaction (RT-PCR)

Total RNA was extracted using TRIzol reagent (Invitrogen™, Waltham, Massachusetts, USA; Cat No. 15596-026). A total of 1 μg of RNA was reverse-transcribed using the PrimeScript RT Master Mix (Takara Bio Inc., Shiga, Japan; Cat No. RR036A) to produce cDNA. The levels of mRNA expression were measured by RT-PCR assays with TB Green PCR Master Mix (Takara, Cat No. 639676, TB Green Advantage qPCR Premix) on a Bio-Rad Real-Time PCR instrument (Bio-Rad, Hercules, CA, USA) using the forward and reverse primers listed in Table S1. Data were analyzed using the ΔΔCt method with β-actin as the reference marker.

### Immunohistochemistry (IHC) Analyses

IHC was applied to paraffin-embedded sections as described in our previous study [9]. Briefly, the sections were dewaxed in xylene, rehydrated in graded ethanols, and immersed in 3% H_2_O_2_ for 10 min to block endogenous peroxidase activity. Antigen retrieval was achieved by immersing the slides in the ethylenediaminetetraacetic acid buffer for 15 min. Non-specific signals were blocked with goat serum for 30 min. Sections were stained with primary antibodies (listed in Table S2) overnight at 4°C in a humid chamber, followed by incubation with HRP-conjugated Streptavidin (R&D Systems, Minneapolis, MN, USA, Cat No. DY998) for 30 min. Finally, a positive signal was generated by incubation with 3,3-diaminobenzidine free base (DAB) (Sigma, Cat No. D5637) for 10 s. The tissue sections were imaged under a fluorescence microscope (BX53, Olympus Corp., Center Valley, PA, USA).

### Quantitative Analyses of Stained Tissue Sections

Fiji software, an open-source image-processing package based on ImageJ (Media Cybernetics, Inc., Rockville, MD, USA), was used to measure the fractional area and integrated optical density (IOD) of the stained slides as reported in our previous study [22]. To summarize, first, the microscopic image of each tissue stained with IHC was transformed into an 8-bit grayscale. Then we created a ‘threshold’ binary image by selecting ‘Image > Adjust > Threshold’ and used the slider to adjust the threshold to match the positive areas. Finally, we called ‘Analyze > Measure’ for the results popup menu with fractional area to get the automatic percentage-stained area value. The OD was obtained using the formula: OD = log (250/Pixel value) under the calibration procedure performed on the 8-bit grayscale images. After processing the threshold of the image as described above, the IOD was measured by clicking ‘Analyze > Measure’ for the integrated density value. DAB staining intensity was determined by mean IOD (mean IOD = IOD/area). The protein levels of each section are represented as an average IOD.

### Quantitative Analyses of Endotoxin Concentration in Plasma

Endotoxin concentrations in plasma were determined using the Bioendo^TM^ End point Chromogenic Endotoxin Test Kit (EC64405S). The data are presented in terms of endotoxin unit (EU) per milliliter.

### Intestinal Barrier Permeability Test

Intestinal epithelial barrier permeability was analyzed by the oral administration of the permeability marker, fluorescein isothiocyanate-dextran (FITC-dextran), 40 kDa (Chondrex, Redmond-Woodinville, Washington, USA; Cat No. 4009). Briefly, 7- to 8-week-old mice were fasted for 4 h and then 40 kDa FITC-dextran was administered by gavage at 20 mL/kg. The mice were then allowed to remain in the cages for 1 h followed by anesthesia. Blood was withdrawn to isolate plasma. Standards were obtained by diluting the FITC-dextran stock solution in phosphate-buffered saline (PBS). Plasma was diluted in an equal volume of PBS (pH 7.4) for analysis and FITC dextran concentrations in plasma were calculated with the help of standard concentrations prepared in PBS at 0.2 µg/mL, 0.4 µg/mL, 0.8 µg/mL, 1.6 µg/mL, 3.1 µg/mL, 6.2 µg/mL, and 12.5 µg/mL. Measurement of the FITC-dextran concentration was carried out on a Cary Eclipse fluorescence spectrophotometer (Agilent, Santa Clara, CA, USA; excitation, 490 nm, emission, 520 nm). Emission signals in mice treated with 40 kDa dextran were excluded from blank values (33% normal mouse plasma in PBS).

### Autistic Behavior Analysis

#### Marble-Burying Test

Repetitive digging behavior was assessed by counting the number of marbles buried for 30 min after placing the BTBR mice in a plastic container [23, 24]. A marble was considered buried when more than 2/3 of its volume was covered by shaved aspen bedding [24]. After the test, the marbles were thoroughly cleaned, and new bedding was used for each mouse.

#### Self-grooming

The self-grooming test was applied as an assay to evaluate repetitive behaviors as previously described [24]. Each mouse was placed in a standard mouse cage. After a 5-min habituation period, each mouse was blindly scored by independent observers for 10 min for the time spent grooming all of its body parts. Full-body grooming or face-wiping, and scratching/rubbing of the head and ears were all defined as self-grooming behaviors. The areas of the cage were cleaned with water followed by 70% ethanol after each test.

#### Open-Field Task (OFT)

The OFT was used to assess psychomotor outcomes and exploratory behaviors [25]. Each animal was individually placed in the central zone of the open field and video-recorded for 20 min with an overhead camera. Distance moved and time spent in the central zone were analyzed by TSE systems (VideoMot2, Germany). A 70% alcohol dissipation was used each time before initiating a new test.

#### Elevated Plus Maze (EPM)

The elevated maze is widely used for testing anxiety behaviors [25, 26]. The specific process was consistent with that reported in most studies [27]. The mouse was placed in the center with the nose pointing at one open arm and was allowed to move freely about the maze for 10 min. The time spent in the open arms was recorded by a video camera attached to a computer and these measurements served as an index of anxiety-like behavior. Finally, the results were calculated by the Image EP program. After each trial, all arms and the central area were cleaned with alcohol to prevent a bias based on olfactory cues.

#### Three-Chamber Test

The three-chamber test was applied to evaluate social preference in the form of general sociability and interest in social novelty in rodent models of autism disorders [28, 29]. As previously described [30], a mouse was habituated to the central chamber of a clear box, which was divided into a three-chamber apparatus, for 5 min, and was allowed to freely explore the chambers for another 10 min.

In the sociability test, a social stimulus (an age- and sex-matched unfamiliar mouse, ‘stranger 1’) was introduced into one cup of the box; the non-social stimulus was referred to as the empty cup.

In the social novelty preference test, a second unfamiliar mouse (‘stranger 2’), was placed in the empty cup located opposite the social stimulus, as in the previous test. The time spent interacting with each cup was recorded by two independent observers. The three-chambered apparatus was cleaned with water followed by 70% ethanol after each test.

### Statistical Analysis

All data are presented as the mean ± SD for each group. Statistically significant differences between groups were analyzed using GraphPad Prism 7 software version (GraphPad, San Diego, CA). Pearson’s correlation was applied to assess relationships between autistic behaviors and intestinal barrier permeability. Student’s two-tailed unpaired *t*-test was used to determine statistically significant differences between experimental groups. *P* values <0.05 were considered to be statistically significant.

## Results

### BTBR Mice Have Gut Barrier Dysfunction Compared to B6 Mice

**Fig. 2:**
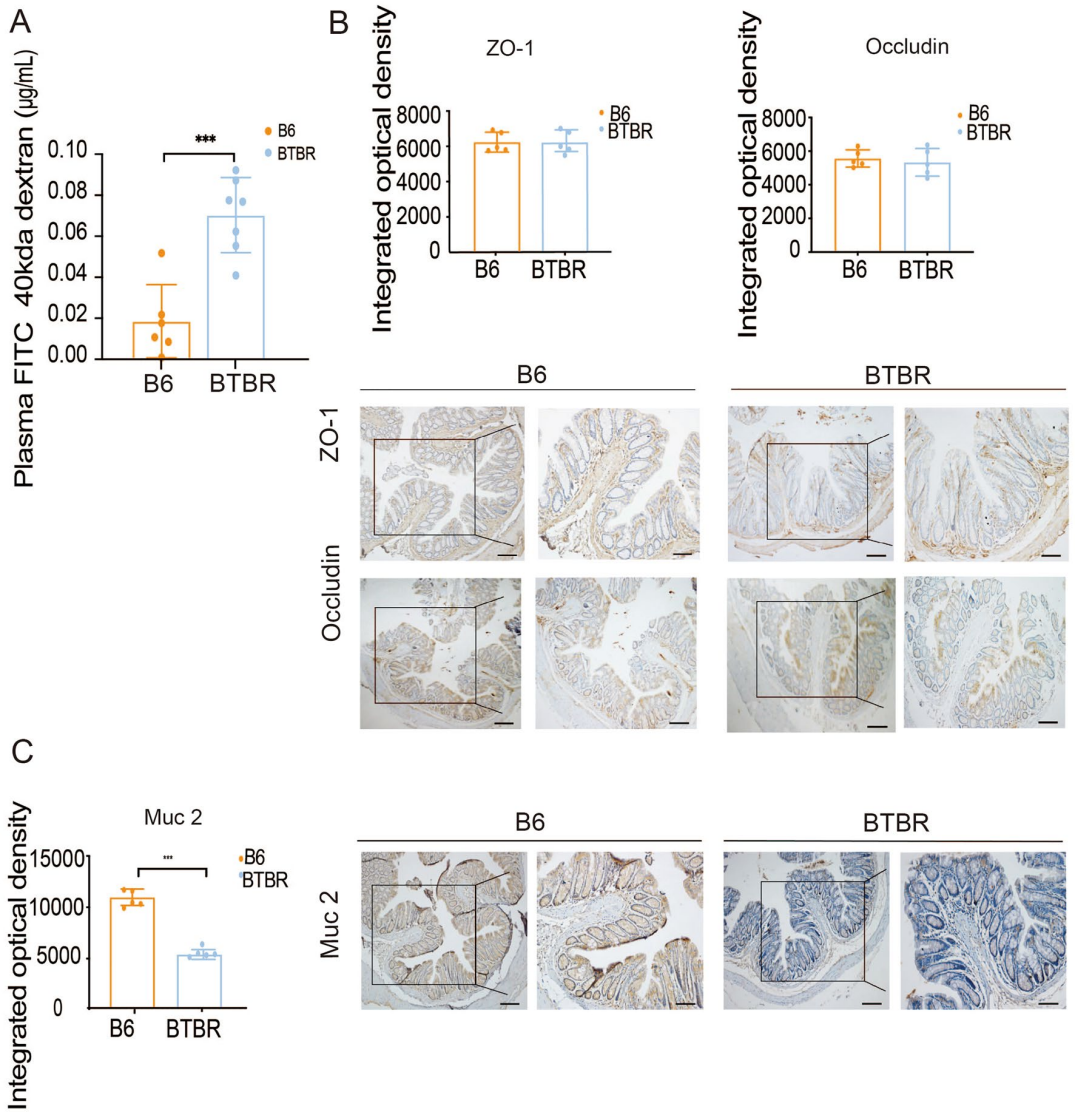
Intestinal barrier dysfunction in BTBR T^+^tf/J mice. **A** FITC-dextran plasma concentrations in C57BL/6 (B6) and BTBR T^+^tf/J (BTBR) mice (*n* = 6–7 per treatment). **B**, **C** Immunohistochemical staining and RT-qPCR analysis of tight junction- and mucus layer-related genes of the intestinal barrier from B6 and BTBR mice (*n* = 5 per treatment). Data are shown as the mean ± SEM and were analyzed by two-tailed unpaired Student’s *t* test. *P <0.05, **P <0.01, ***P < 0.001. Images were captured by confocal microscopy. Scale bars, 40 μm (left graph) and 20 μm (the inset of the left graph). The immunostaining analyses compare the gene expression levels. B6, C57BL/6 mice; BTBR, BTBR T^+^tf/J mice; ZO-1, zonula occludens 1; Muc 2, Mucin 2. RT-qPCR, reverse transcription-quantitative polymerase chain reaction.

Similar to humans suffering from autism [31], BTBR mice (autism model) also have a “leaky” intestinal epithelium (Fig. 2). To characterize epithelial permeability in the intestine, we measured the efficacy of macromolecular diffusion across the epithelium *in vivo* (40 kDa FITC flux) and found a dramatic increase in epithelial permeability in BTBR mice (Fig. 2A). The FITC flux concentration in plasma of BTBR mice was more than double that in controls at the 60-min time point. The macromolecular permeability of intestinal epithelium is largely dependent on the proper function of TJs and the mucus layer. Despite an increase in FITC flux, we failed to find substantial alterations in the expression levels of TJ proteins [zonula occludens-1 (ZO-1) and occludin] (Fig. 2B). Interestingly, the expression of Muc2, a gene encoding mucin 2, which is the major constituent of the mucus layer, was down-regulated in the large intestine of BTBR mice (Fig. 2C), which is consistent with the finding of Golubeva *et al.* [32].

### 2% DSS Induces Gut Leakage in BTBR Mice

**Fig. 3:**
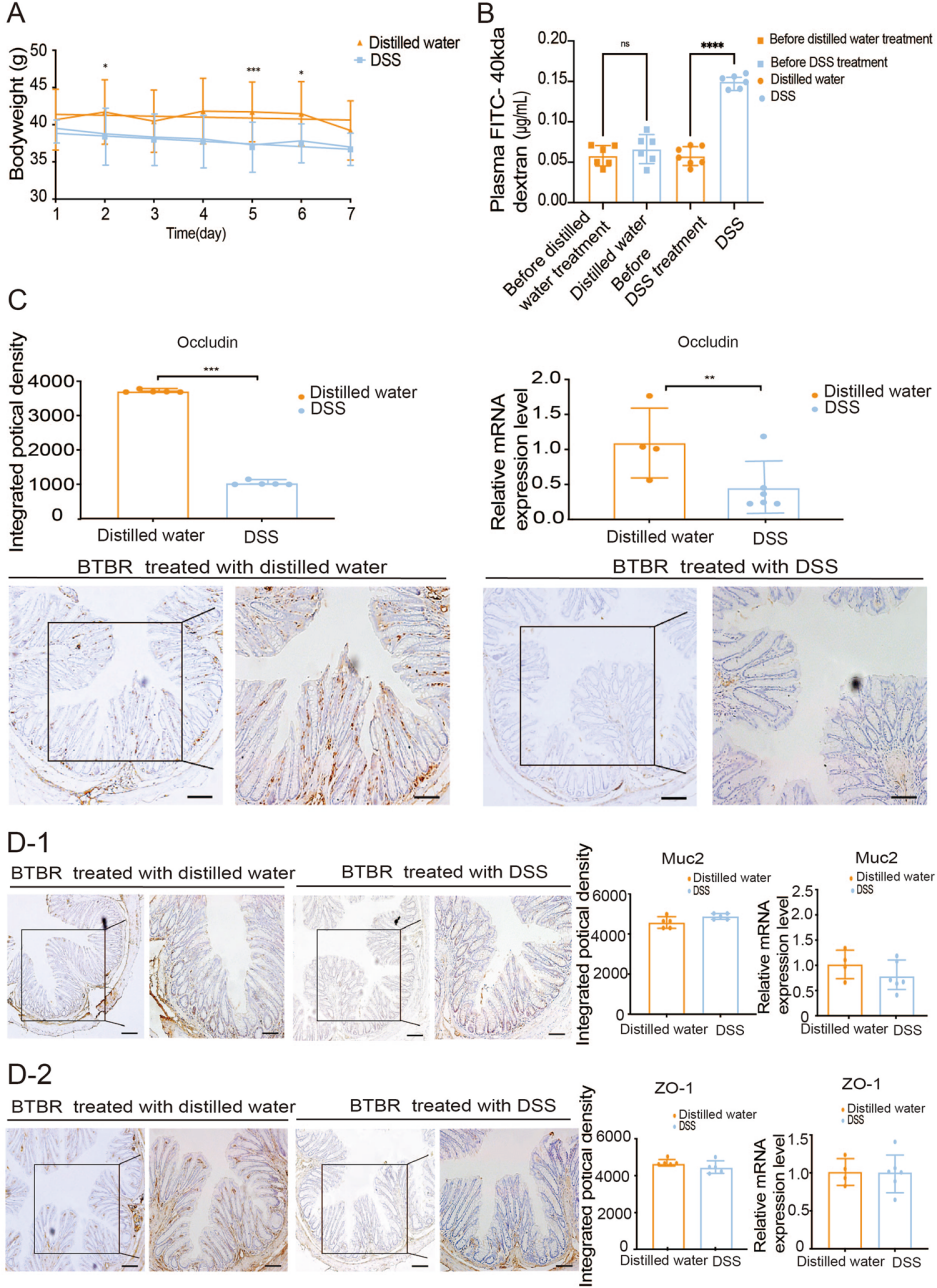
Administration of DSS specifically induces epithelial barrier dysfunction in BTBR mice. **A** Body weights of mice from day 0 to day 7 (*n* = 12–13 per treatment) and recorded every two days. **B** FITC-dextran levels in the plasma of BTBR mice receiving DSS treatment or distilled water (*n* = 6–7 per treatment). **C**, **D** Tight junction- and mucus layer-related gene expression in the colon. ZO-1, Occludin, and Muc 2 were analyzed with immunohistochemistry staining (*n* = 5 per treatment) and RT- qPCR (*n* = 4 for distilled water, *n* = 6 for DSS treatment). Images captured by confocal microscopy. Scale bars, 40 μm (left graph) and 20 μm (the inset of the left graph). Immunostaining analyses compare the gene expression levels. DSS, DSS-treated BTBR mice; Distilled water, distilled water-treated BTBR mice. Data are shown as the mean ± SEM. *P <0.05, **P <0.01, ***P <0.001, two-tailed unpaired Student’s *t* test. ZO-1, zonula occludens 1; Muc 2, Mucin 2. RT-qPCR, reverse transcription-quantitative polymerase chain reaction.

Gut leakage was induced in male BTBR mice by the addition of 2% DSS to the drinking water for 7 days. To avoid affecting the normal behavior of the mice, care was taken not to introduce sustained diarrhea and rectal bleeding owing to the treatment. The maximum weight loss did not exceed 7% of the total body weight after DSS treatment (Fig. 3A; the shift from the lowest average weight on the seventh day to baseline was 6.13%). Histological analysis revealed differences in intestinal barrier permeability in mice of the DSS group, and damage of intestinal epithelium was seen in DSS-treated BTBR mice. An *in vivo* permeability assay, using 40 kDa FITC-dextran, revealed that the intestinal barrier permeability was much higher in DSS-treated BTBR mice than in distilled water-treated mice (Fig. 3B), with increased concentration of dextran in plasma. An altered expression of TJs and mucus proteins that are closely associated with epithelial barrier integrity [17] explains this phenomenon. Compared with distilled water-treated BTBR mice, DSS-treated mice demonstrated decreased expression of occludin in the colon samples, as shown by IHC and RT-PCR data (Fig. 3C, D).

### Increased Gut Barrier Permeability Aggravates Autistic Behavior in BTBR Mice

**Fig. 4:**
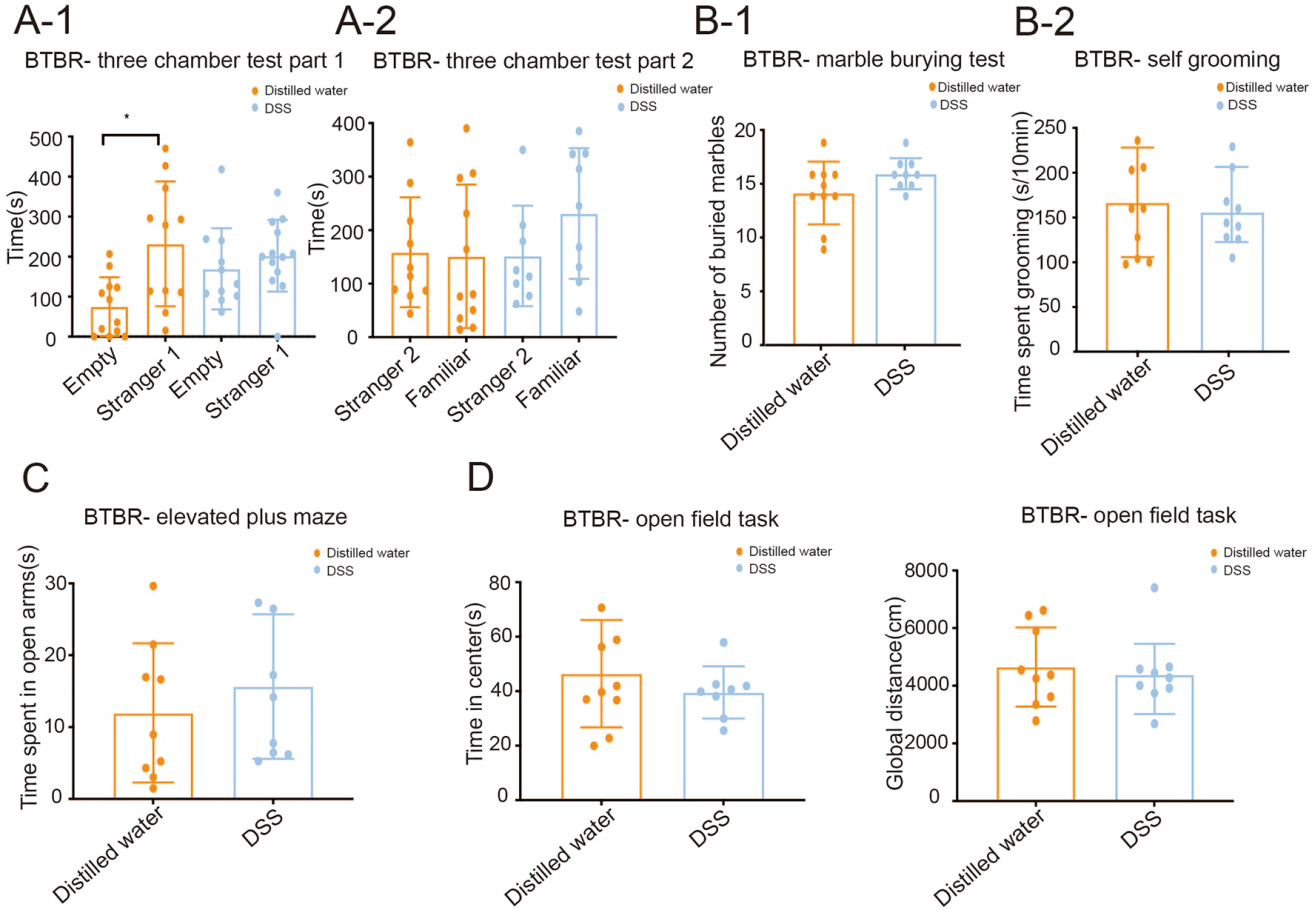
Effects of DSS on the behavioral performance of BTBR mice. In three-chamber tests, mice given distilled water spend significantly more time in the ‘stranger 1’ chamber and indicate more social proximity (**A**). DSS treatment does not change repetitive behaviors (**B**) or anxiety (**C**, **D**) behaviors. DSS, DSS-treated BTBR mice; Distilled water, distilled water-treated BTBR mice; Data are shown as the mean ± SEM. *P <0.05, **P <0.01, ***P <0.001, two-tailed unpaired Student’s *t* test (*n* = 8–13 per treatment).

There was an apparent effect of intestinal permeability on the time spent by the mice investigating sociability and novelty. Compared to DSS-treated mice, distilled water-treated mice spent more time interacting with the ‘stranger 1’ mouse than the empty object; this finding was notwithstanding the difference in time spent between ‘stranger 1’ and ‘stranger 2’ (Fig. 4A-1, A-2). These results indicate that increased intestinal barrier permeability causes less preference for social proximity. But there was no interaction between gut permeability and repetitive (Fig. 4B) or anxiety (Fig. 4C, D) behaviors. There were no significant differences between DSS and distilled water-treated mice regarding the number of marbles buried (Fig. 4B-1). Furthermore, the self-grooming test demonstrated that the leaky gut did not have a major effect on repetitive behavior (Fig. 4B-2). In addition, increased intestinal permeability had no effect on the amount of time spent in the open arms (Fig. 4C). Distance or time traveled in the open field was also measured in the open-field task test (Fig. 4D). It was interesting to note that DSS-treated BTBR mice traveled no further than their controls. Furthermore, no significant differences were found in the time spent in the central area between DSS- and distilled water-treated BTBR mice.

### Metformin Improves Intestine Barrier Dysfunction *via* Increasing Expression of Mucin 2

Metformin, with a favorable safety profile, is one of the most frequently prescribed drugs worldwide due to the high prevalence of type 2 diabetes (T2D) [33]. First marketed for T2D in 1957, metformin has commanded a significant market share in the ensuing decades and is a mainstay of treatment, with a current prescription rate of 77% in T2D patients [34]. Metformin is now considered to have multiple beneficial effects in humans, other than its use as an antidiabetic drug [35–37]. Our team previously demonstrated that metformin protects against intestinal barrier dysfunction through its effects on intestinal permeability [38]. We chose metformin as a positive stimulant not only because of its protective barrier effect but also because it has no negative effects on the anatomy of the brain and does not activate intracranial TLR4/MyD88/NF-κB signaling pathways resulting in an increase of pro-inflammatory factors (Fig. S1). This is consistent with the conclusion that metformin has a potential neuroprotective effect in neurodegenerative disease reported in the literature [39–41].

**Fig. 5:**
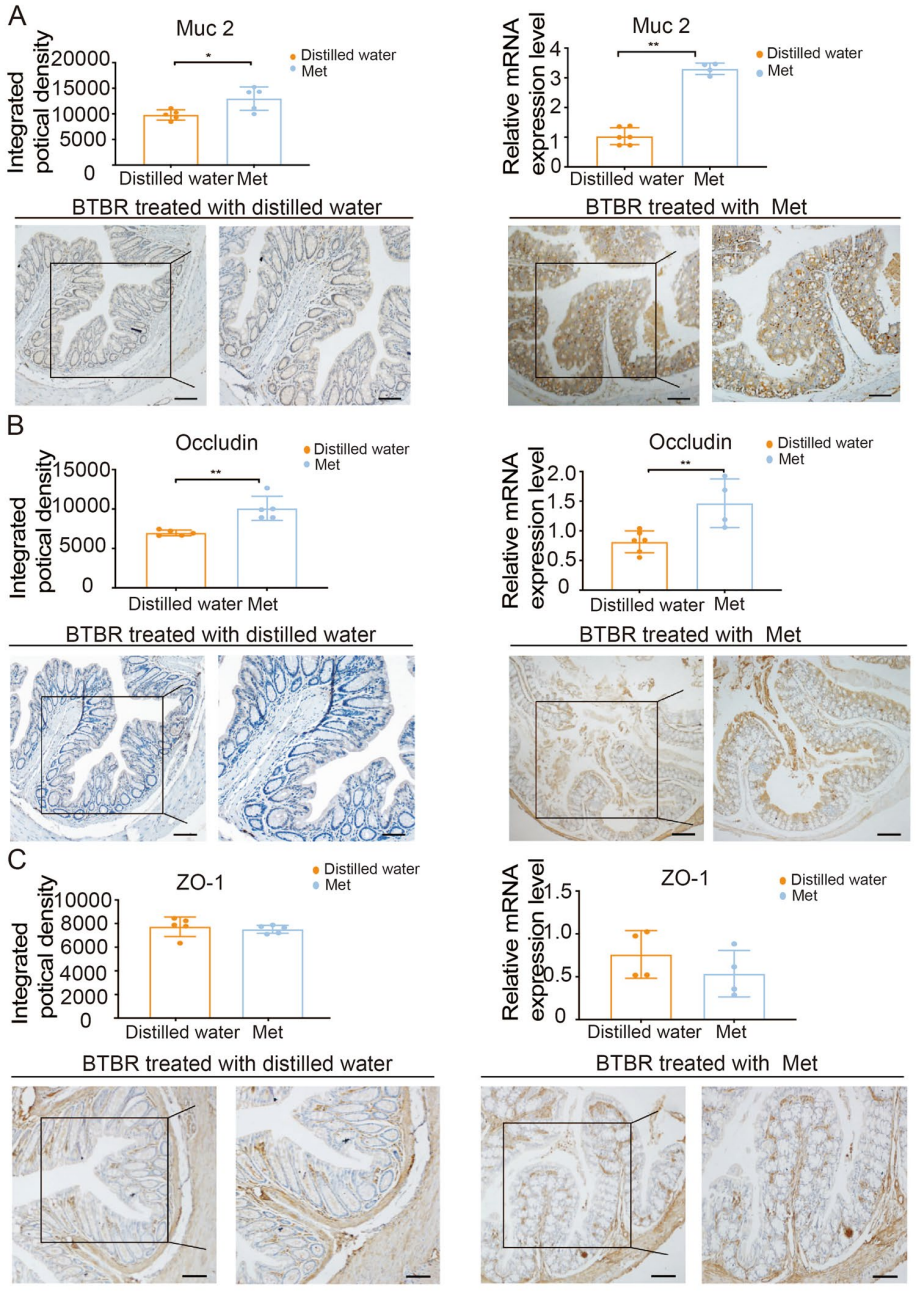
Protective effects of metformin on intestinal barrier dysfunction in Metformin group mice. After 14 days of metformin treatment, immunohistochemistry (*n* = 5) and RT-qPCR (*n* = 4–6) were used to evaluate Muc 2 (**A**), Occludin (**B**), and ZO-1 (**C**) expression. The images were captured by confocal microscopy. Scale bars, 40 μm (left graph) and 20 μm (the inset of the left graph). Data are shown as the mean ± SEM. *P <0.05, **P <0.01, ***P <0.001, two-tailed unpaired Student’s *t* test. Met, metformin-treated BTBR mice; Distilled water, distilled water-treated BTBR mice; ZO-1, zonula occludens 1; Muc 2, Mucin 2. RT-qPCR, reverse transcription-quantitative polymerase chain reaction.

It has been previously demonstrated that the phenomenon of leaky gut exists in BTBR mice with downregulation of mucin 2 expression levels (Fig. 2C). It is surprising that not only was the decrease in the expression levels of mucus protein—Muc 2 reversed (Fig. 5A), but occludin expression also significantly increased, when mice were administered metformin (400 mg/kg) by oral gavage (Fig. 5B). However, there were no significant differences in the TJs (ZO–1) between metformin-treated and distilled water-treated BTBR mice (Fig. 5C).

**Fig. 6:**
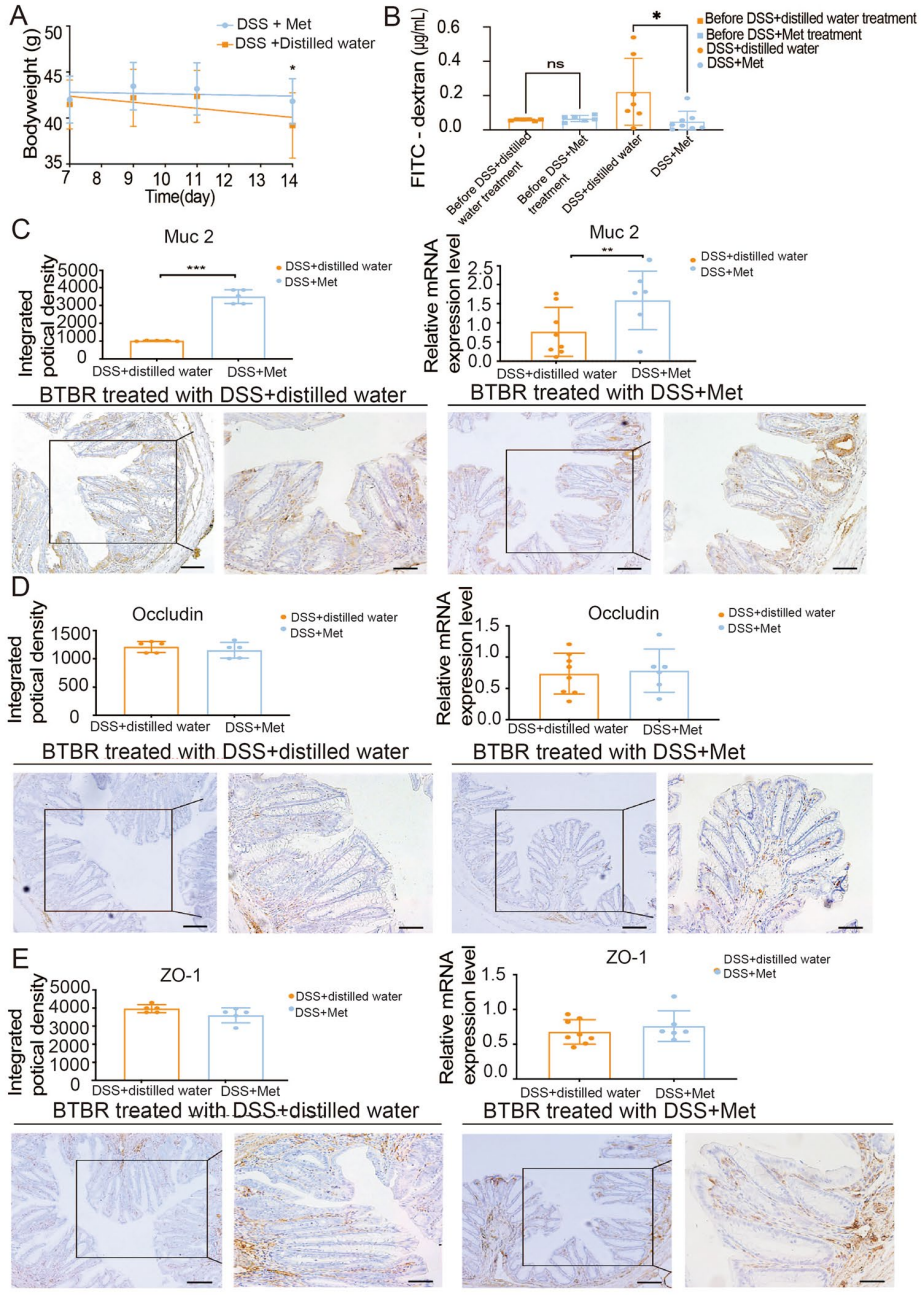
Protective effects of metformin on intestinal barrier dysfunction in Metformin plus DSS group mice. (**A**) and (**B**), changes in body weight during 7 days (*n* = 10–13 per treatment) and plasma FITC–40 kDa dextran concentration (*n* = 6–8 per treatment) are shown in the Metformin plus DSS group. Muc 2 (**C**), Occludin (**D**), and ZO-1 (**E**) mRNA expression and protein levels in the colonic mucosa of the Metformin plus DSS group (*n* = 6–8 per treatment for RT-PCR, *n* = 5 per treatment for immunohistochemistry).The images were captured by confocal microscopy. Scale bars, 40 μm (left graph) and 20 μm (the inset of the left graph). Data are shown as the mean ± SEM. *P <0.05, **P <0.01, ***P <0.001, two-tailed unpaired Student’s *t* test. BTBR mice received 2% DSS in the first week followed by metformin or distilled water treatment for the next week (DSS+Distilled water and DSS +Met mice, respectively). ZO-1, zonula occludens 1; Muc 2, Mucin 2. RT-qPCR, reverse transcription-quantitative polymerase chain reaction.

To further confirm our hypothesis, we administered 400 mg/kg of metformin to BTBR mice for another 7 days after DSS treatment to confirm its role in intestinal barrier protection. As we expected, metformin treatment after DSS administration restored the loss in body weight (Fig. 6A) and significantly reduced the increased 40-kDa FITC-dextran concentration in plasma (Fig. 6B). Metformin induced significant changes in barrier function, owing to the dramatic increase in the Mu2 expression levels (Fig. 6C) after metformin exposure in DSS treated-BTBR mice regardless of Occludin and ZO-1 levels (Fig. 6D, E). Based on this evidence, metformin might be capable of reducing gut permeability *via* increased expression of Muc 2 or both occludin proteins.

### Decreased Gut Permeability Alleviates Symptoms of Autism

We have shown that aggravating the disruption of the intestinal barrier can aggravate the social behaviors of autistic mice. To provide a more complete chain of evidence, we demonstrated that reversing intestinal barrier disruption with metformin significantly alleviated repetitive and social behaviors in BTBR autistic mice.

**Fig. 7:**
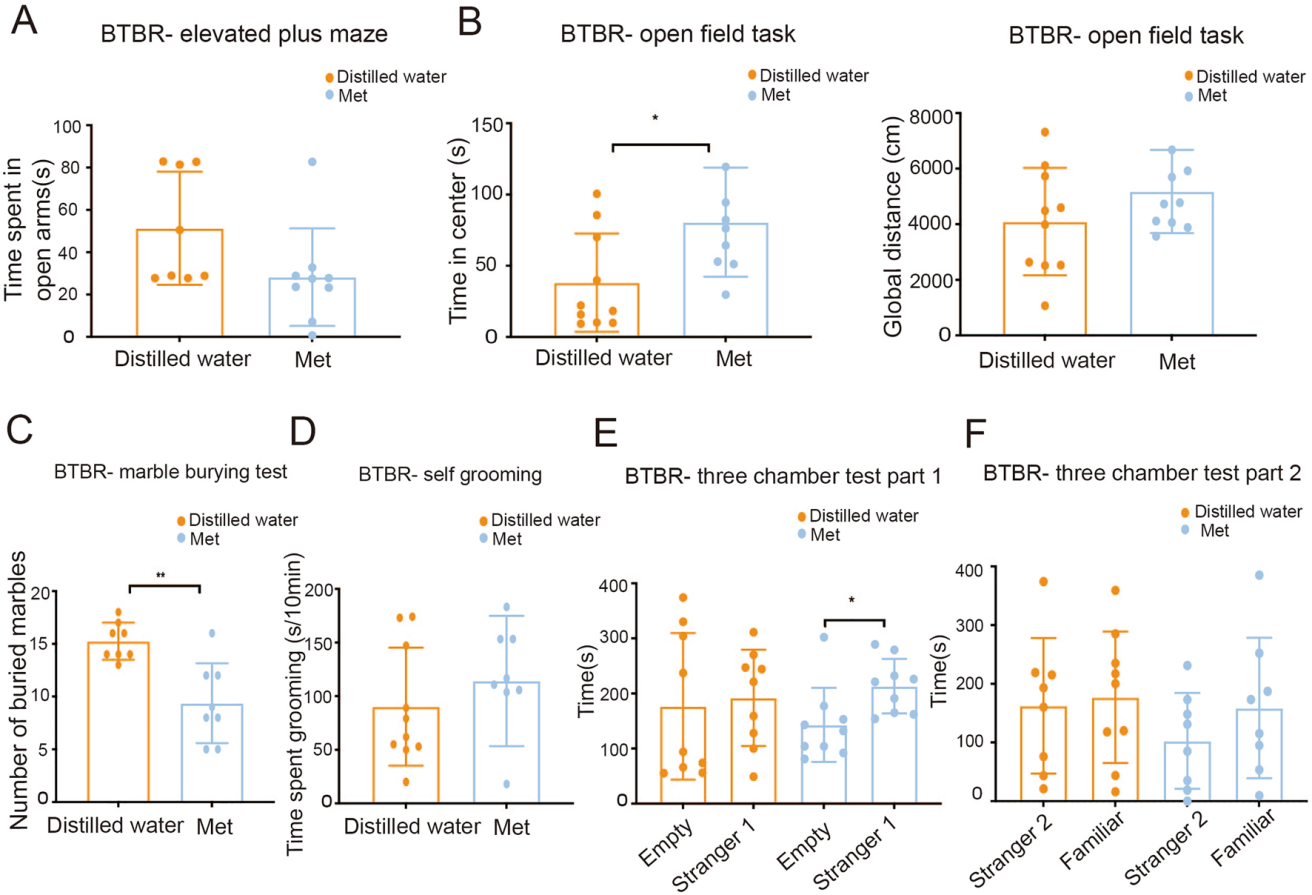
Effects of metformin on the behavioral performance of mice in the Metformin group. In BTBR mice, metformin therapy increases the time spent in the center in the open field test (**B**) but does not affect anxiety-linked behavior in the elevated plus maze test (**A**). Metformin consumption reduces marble burying (**C**) but does not affect self-grooming (**D**). In the three-chamber test, metformin treatment in BTBR mice spend significantly more time in the ‘stranger 1’ chamber and indicate more proximity (**E**). Met, metformin-treated BTBR mice; Distilled water, distilled water-treated BTBR mice. Data are shown as the mean ± SEM. *P <0.05, **P <0.01, ***P <0.001, two-tailed unpaired Student’s *t* test (*n* = 8–10 per treatment).

The EPM and OFT tests were used to investigate whether high levels of anxiety-like responses were present [25–27]. Fig. 7A shows the time spent on the open arms of the plus-maze in the metformin group. In the EPM test, we noted that metformin in autistic animals did not have a significant effect on the time spent in the open arms compared to that spent by distilled water-treated mice. The OFT is another method widely used to measure anxiety-related behaviors [42]. Although metformin did not considerably increase the total distance traveled in 30 min, it significantly increased the time spent in the center by BTBR mice compared with that spent by distilled water-treated mice (Fig. 7B). These results suggest that metformin improves autistic anxiety behavior in the OFT *via* repairing the gut barrier to a physiological state.

Repetitive behaviors were assessed by marble-burying and self-grooming tests, which reflect ethologically normal, stereotypical behaviors in rodents [43]. After metformin treatment in BTBR mice, the number of buried marbles was lower than in distilled water-treated mice (Fig. 7C). But self-grooming behavior did not significantly differ between the metformin- and distilled water-treated mice (Fig. 7D). This indicates that metformin improves repetitive behaviors, as assessed using the marble-burying test.

Finally, we conducted social behavioral tests (Fig. 7E, F). With respect to sociability, metformin-treated BTBR mice spent more time exploring the chamber with the ‘stranger’ mouse than they spent in the empty chamber when compared to the distilled water-treated mice (Fig. 7E); metformin administration in BTBR mice increased the social proximity behavior. Furthermore, animals in the metformin group did not show any particular preference for social novelty behavior (Fig. 7F). Metformin management in BTBR exhibited a similar time spent between familiar and ‘stranger 2’ mice.

**Fig. 8:**
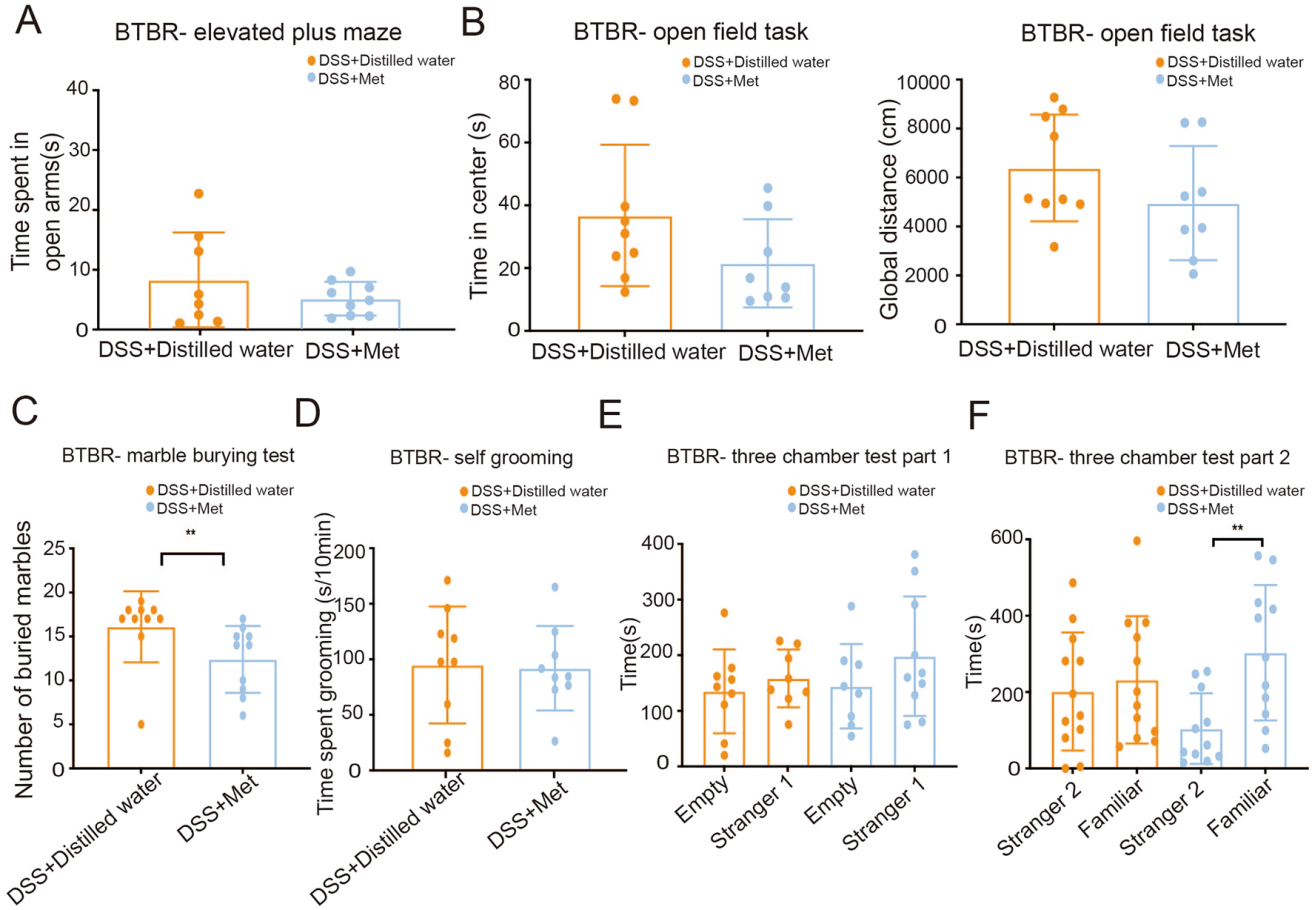
Effects of metformin on the behavioral performance of mice in the Metformin plus DSS group. The BTBR mice receiving metformin management after DSS treatment show an increased preference for the familiar mouse chamber (**F**), indicating more social memory. BTBR mice received 2% DSS in the first week followed by metformin or distilled water treatment for the next week (DSS+Distilled water and DSS +Met mice). Data are shown as the mean ± SEM. *P <0.05, **P <0.01, ***P <0.001, two-tailed unpaired Student’s *t* test (*n* = 8–12 per treatment).

Although the same behavioral pattern cannot be ascribed to the autistic mice in the metformin plus DSS group (Fig. 8A–F), an overall trend of improvement in autistic behavior was consistently seen. Although metformin intervention after DSS treatment did not affect anxiety-related behavior (Fig. 8A, B) and social proximity (Fig. 8E) or social novelty performance (Fig. 8F), it caused a significant decrease in the number of buried marbles (Fig. 8C) and an increase in time spent exploring the familiar mouse chamber (Fig. 8F), which indicated that these mice demonstrated fewer repetitive and more social memory behaviors than the BTBR mice that received DSS and distilled water treatment.

### The TLR4–MyD88–NF-κB Signaling Pathway May Play a Key Role in Aggravating Autistic Behavior

**Fig. 9:**
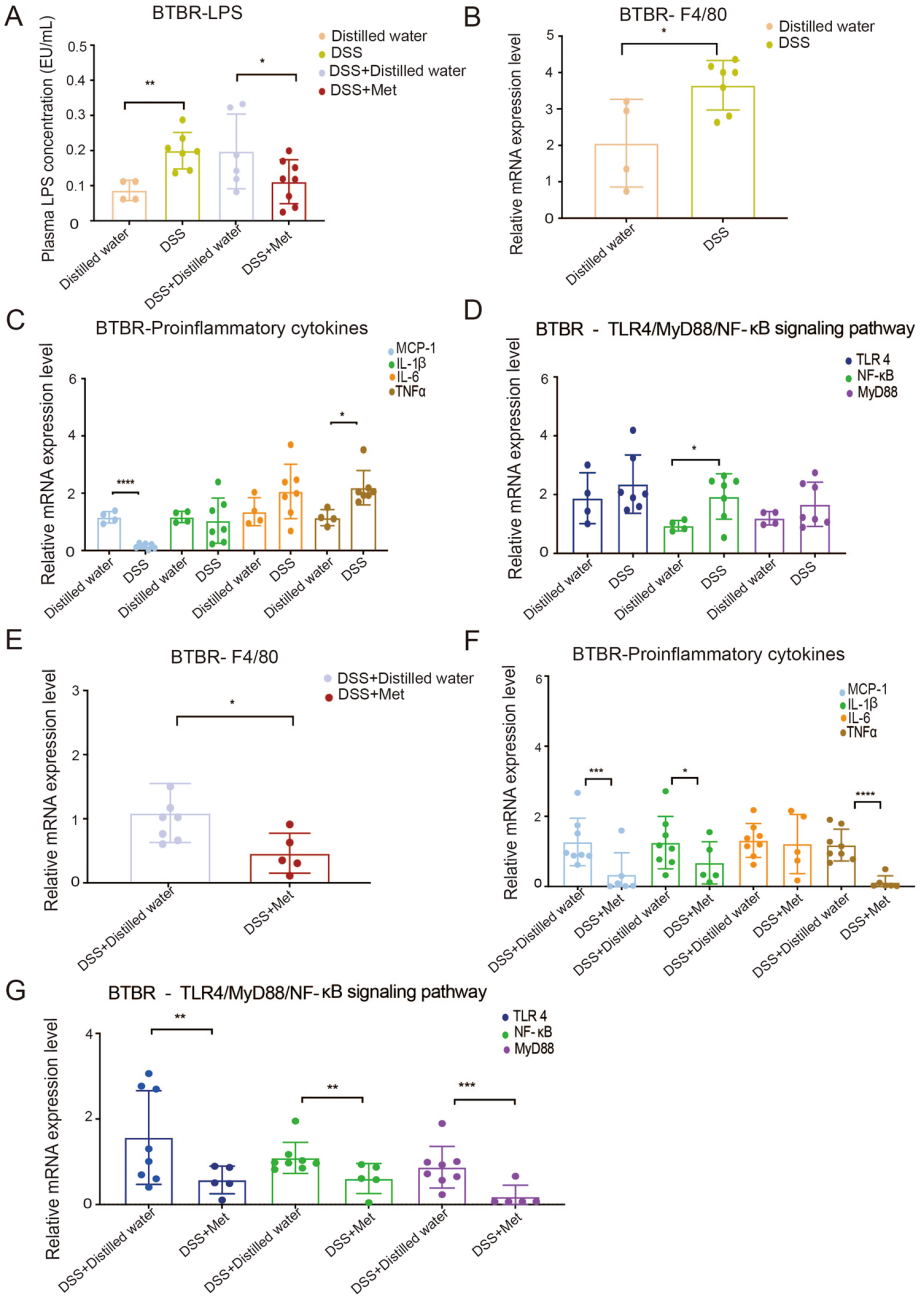
Metformin regulates intracranial inflammation through TLR 4/NF–κB/MyD88 signaling *in vitro*. Metformin lowers endotoxin levels in plasma (**A**) and suppresses activation of the TLR 4/NF-κB/MyD88 signaling pathway (**G**) in the brain of BTBR mice treated with DSS; the mRNA levels of the major macrophage marker F4/80 (**B, E**), signaling pathway components TLR4, MyD88, and NF-κB (**D, G**), and downstream pro-inflammatory cytokines TNFα, MCP-1, IL-6, and IL-1β (**C, F**) measured by RT-qPCR. DSS, DSS-treated BTBR mice; Distilled water, distilled water-treated BTBR mice; BTBR mice received 2% DSS in the first week followed by metformin treatment or distilled water for the next week (DSS+Distilled water and DSS+Met mice). RT-qPCR, reverse transcription-quantitative polymerase chain reaction. Data are shown as the mean ± SEM (*n* = 4 for the mice receiving distilled water; *n* = 7 for the mice receiving DSS treatment; *n* = 5–8 or 6–8 for mice receiving metformin treatment or distilled water after DSS consumption). *P <0.05, **P <0.01, ***P <0.001, two-tailed unpaired Student’s *t* test.

Endotoxin, a type of LPS, is elevated in patients with autism [28, 44]. The central pathway by which LPS leads to an inflammatory environment in the brain causing neurodegeneration in patients with autism is as follows: gut endotoxins may enter the blood due to a leaky gut and subsequently enter the brain tissue by crossing the blood-brain barrier and cause brain inflammation *via* activation of the TLR4–MyD88–NF-κB transcriptional pathway in macrophages to induce pro-inflammatory cytokines, such as tumor necrosis factor α (TNF-α), monocyte chemoattractant protein-1 (MCP-1), interleukin-1 (IL-1), IL-6, and IL-1β [28, 45, 46]. The LPS levels in plasma were higher after intestinal barrier damage as exhibited in DSS-treated BTBR mice (Fig. 9A). To investigate whether downstream cytokines are regulated by the TLR4–MyD88–NF-κB signaling pathway in macrophages stimulated by gut-derived LPS, we measured the primary macrophage marker F4/80 and key molecules for the signaling pathway (TLR4, MyD88, and NF-κB) in brain tissue. We noted that the LPS challenge significantly increased the expression of F4/80 and NF-κB in DSS-treated BTBR mice (Fig. 9B, D). Moreover, the expression of the downstream pro-inflammatory factor TNF-α was also increased correspondingly (Fig. 9C). Although there was no statistical difference in the expression of MyD88 and TLR4 between the two different treatments, DSS-treated BTBR mice showed an increasing trend of NF-κB compared to distilled water-treated mice (Fig. 9D).

Metformin administration in DSS-treated BTBR mice repressed macrophages (Fig. 9E) and excessive release of inflammatory factors (MCP-1, IL-1β, and TNFα) (Fig. 9F). It also resulted in infiltration of the cerebral cortex by these inflammatory factors, which was partly attributable to inhibition of the TLR4–MyD88–NF-κB signaling pathway as demonstrated by the downregulation of expression of all three key molecules: TLR4, MyD88, and NF-κB (Fig. 9G).

To establish the specific brain areas where these important pro-inflammatory cytokines are expressed, we added immunohistochemical studies (Fig. S2). Our findings revealed that these essential inflammatory factors were expressed in the dorsomedial cerebral cortex and the CA1 substructure of the hippocampus and that changes in protein levels measured by IHC corresponded to the changes in mRNA levels measured by RT-PCR.

## Discussion

Few studies have been performed that determine the role of abnormal gut permeability in the neuropsychiatric manifestations of ASD. In this study, we aimed to clarify the effect of “leaky gut” on the various autistic behavioral phenotypes using BTBR mice (an autism model). Our study provided evidence that increased intestinal barrier permeability may make a vital contribution to the pathological changes in autism; it induces a gut-derived endotoxin to transfer to the brain and create a pro-inflammatory environment *via* activation of the TLR4–MyD88–NF-κB signaling pathway. Listed below are the main findings of the study:

(1) Administration of metformin to BTBR mice caused a dramatic decrease in repetitive behaviors measured by marble burying, which may be a consequence of intestinal barrier impairment;

(2) Loss of function of the intestinal barrier caused a decreased tendency for social proximity in autistic mice; administration of metformin reversed this phenomenon;

(3) LPS levels in plasma were higher in DSS-treated autistic mice than in mice receiving metformin treatment;

(4) The expression levels of the pro-inflammatory cytokines MCP-1, IL-1β, and TNFα and the degree of macrophage infiltration declined after metformin treatment, and expression of the upstream regulatory molecules TLR4, MyD88, and NF-κB also decreased.

Consequently, we postulate that restoring the gut barrier with metformin could be an effective method to improve autistic behavior in adult mice with autism.

ASD refers to a broad range of conditions characterized by cognitive inabilities, repetitive behavior patterns, and restricted social interaction and communication [1]. Gastrointestinal disorders are the most common problems in patients with ASD. Despite their prevalence, they are often overlooked [47]. Leo Kanner, a Boston physician, who first described autism, reported in an article published in 1943 that most of the children with autism had more difficulty in eating or feeding at a young age, i.e., they had issues with food selectivity, food refusal, and poor oral intake compared to healthy children, which supported the connection between autism and the gut, thereby affecting the pathophysiology of ASD [48]. Extensive studies have identified the dynamic and bidirectional interaction between the gut and brain along the “gut–brain axis”. Different metabolites of intestinal origin have been reported in the blood and brain of autistic children, such as short-chain fatty acids, indoles, LPS, and serotonin [49–51]. This explains how the leakage of intestinal metabolites into the brain may trigger autistic behavioral responses. Thus, damage to the gut barrier may act as a key bridge in the development of autism.

Impairment of intestinal the barrier in autism has been reported [52]. Several investigations have found that people with autism have a damaged intestinal barrier: D’Eufemia *et al.* reported that an altered intestinal permeability was found in 9 of 21 (43%) autistic patients based on the most traditional method for ascertaining gut permeability, the lactulose: mannitol test (comprising two different sugars given orally and output measured in urine), but in none of the 40 controls [18], and this was among the first evidence for a leaky gut in autism. Magistris *et al.* in 2010 reported on the presence of abnormal intestinal permeability (leaky gut) in over a third of a cohort of children diagnosed with autism (36.7%) [17]. More than that, the studies on leaky gut and autism do not stop there. Previous studies found decreased intestinal TJ claudin in ASD patients [16, 53]. In Esnafoglu’s study, serum zonulin, a measure of increased intestinal permeability, was considerably higher in autistic children than in healthy controls [54]. These are consistent with our results. The efficacy of macromolecular diffusion across the epithelium *in vivo* (40-kDa FITC flux) was measured to characterize the differences in intestinal epithelial permeability between BTBR and B6 mice. As previously reported [32], increased intestinal permeability in BTBR mice caused a much higher FITC flux than in B6 mice. Consistent with this, we found molecular dysfunction of the intestinal barrier. Despite failing to find alterations in TJs (ZO-1, occludin), the expression of Muc2, a constituent of the main epithelial mucus layer in BTBR mice, was significantly lower. These findings strongly suggested that the intestinal barrier may play an irreplaceable role in the development of autistic behavior.

Many studies have indicated that metformin might have a beneficial effect on neurodegenerative diseases. Chronic treatment with metformin for 24 weeks significantly improves the cognitive and depressed performance in depressed patients with T2DM [55]; Metformin treatment rescues spatial memory impairment and neuronal death in the hippocampus and promotes hippocampal neurogenesis in a mouse model of Alzheimer’s disease [56]; Metformin treatment *in vitro* increases the neuronal activation of partitioning defective 1, a family of Ser/Thr kinases playing a key role in synaptic plasticity and neuroinflammation in functional recovery from traumatic brain injury [57]. There is no doubt that metformin has the potential to improve the cognitive and behavioral deficits shown in ASD models. Although metformin has been reported to improve autistic behavior in mouse pups [58], we are the first to report the influence of metformin in adult mice in alleviating autistic behavioral phenotypes by protecting the integrity of the intestinal barrier; this offers the hope of a cure for autism in adults. To confirm our hypothesis, we applied positive and negative tests to explore the alteration in intestinal barrier permeability and its impact on autistic behavior by further disrupting and repairing the intestinal barrier. To avoid excessive intestinal inflammation that could have affected the normal activities of mice, 2% DSS was administered only for a week, resulting in a minimal loss in weight ( 7% of the total body weight, Max weight = 38.25 ± 4.781 g; Min weight = 35.07 ± 4.728 g) and no blood in stool; however, diarrhea was observed. The expression of occludin in the intestinal epithelial decreased and the concentration of dextran in plasma increased correspondingly, which indicated that the addition of DSS serves as a negative regulator of gut permeability. As a positive stimulus, metformin was chosen to restore the “leaky gut”; as it has been demonstrated to protect against intestinal barrier dysfunction in previous studies [51] and it does not contribute to the development of an inflammatory environment in the brain. Our results demonstrated that metformin not only had a beneficial effect in preserving the intestinal barrier integrity of BTBR mice *via* upregulating the expression of the main TJ, occludin, and the mucus barrier molecule, Muc 2, in a physiological state, but it also protected mice from the intestinal barrier damage caused by 2% DSS. Administrating metformin to DSS-treated BTBR mice showed a significantly reduced loss in body weight compared to those treated with DSS only. Consistent with these findings, immunohistochemical examination showed higher protein Muc 2 expression in DSS+Met mice; in addition, RT-PCR results provided strong evidence to support this finding.

It is noteworthy that the permeability of the intestinal barrier changed along with the autistic behavior of BTBR mice. Repetitive behaviors are considered to be a core diagnostic feature of autism [59]. Repetitive behaviors in patients with autism are characterized by jumping, spinning, head tilting, nodding, and arm or hand flapping [35]. In rodents, one way of assessing such behaviors is through marble-burying and self-grooming tests, as these reflect ethologically normal and stereotypical behaviors in rodents [43]. Although the damage to the intestinal barrier with DSS showed no differences in the number of buried marbles, administration of metformin to BTBR mice or DSS-treated BTBR mice, dramatically decreased the number of marbles buried compared to those buried by distilled water-treated BTBR mice or both DSS and distilled water-treated BTBR mice. However, changes in the integrity of the intestinal barrier had no effect on self-grooming behavior. These results suggested that repetitive behaviors measured by marble burying may be a consequence of intestinal barrier impairments.

The EPM and OFT are routinely used to study anxiety-related behavior in mice [28]. When anxiety-related behavior was analyzed, the increased intestinal barrier permeability induced by DSS had no effect on the EPM and OFT tests. Metformin was administered to BTBR and DSS-treated BTBR mice and these mice neither showed a significant increase in the time spent in open arms compared to distilled water-treated and both DSS and distilled water-treated BTBR mice in the EPM test. As another method to measure anxiety in animals, similar levels of locomotor activity in the OFT test were exhibited in the metformin group and the metformin plus DSS group. However, the exploratory behaviors were improved in BTBR mice after receiving metformin; they spent much more time in the center than the BTBR mice that received distilled water.

Our study demonstrated that loss of intestinal barrier function might cause a deficit in social interaction and recognition. Unlike DSS-treated BTBR mice, distilled water-treated mice displayed a greater preference for the ‘stranger 1’ mouse. This result indicated that an increased intestinal barrier permeability results in a reduced preference for social proximity. As expected, restoring the gut barrier function using metformin in BTBR mice reversed this phenomenon, and consequently, mice showed more interest in interaction with the ‘stranger 1’ than with the empty chamber.

Repair of intestinal barrier permeability by metformin did not have any apparent effect on the social novelty behavior of autistic mice; however, metformin-treated BTBR mice after DSS intervention did demonstrate more interest in communicating with familiar mice. Therefore, the repair of intestinal barrier permeability tended to increase social memory, which has the potential to improve a vital behavioral defect in autistic mice, as seen by the absence of a time delay for recognizing the familiar mouse [60].

As a result, alterations in the intestinal barrier appear to be linked to autistic behavioral changes in BTBR mice. To assess the causal association between alleviating autism-like behaviors and repairing the intestinal barrier, we conducted a correlation study on significantly improved autistic behaviors and changes in intestinal barrier permeability following intervention (Fig. S3). In the three-chamber test, BTBR mice spent less time with ‘stranger 1’ as their gut barrier permeability increased (Fig. S3A) and more time with ‘stranger 1’ after the permeability decreased with metformin treatment (Fig. S3B3). This shows that social deficit behaviors in autism can be improved by lowering intestinal barrier permeability. And as intestinal barrier permeability declined, the number of marbles buried declined as well (Fig. S3B1), correlating with an increase in time spent in the center of the OFT (Fig. S3B2), which shows that reduced intestinal barrier permeability may improve repetitive stereotypical and anxiety-related behaviors in autistic mice. All these results suggested that gut leakage might be the etiology of autism and improvement in intestinal barrier dysfunction may be a potential target for relieving the symptoms of ASDs.

Finally, we sought to further understand the underlying mechanisms that increased the permeability of the intestinal barrier contributing to autism. Studies have shown evidence of increased gut metabolites and serum endotoxin levels in patients with ASD, and this offers a mechanism by which a “leaky gut” could play a role in this neurodevelopmental disease [10, 61, 62]. Among the potential mechanisms by which absorption of LPS from the gut lumen affects autistic behaviors, activation of the innate immune system in the brain owing to circulating pro-inflammatory cytokines has been demonstrated in many studies [63]. LPS can act upon TLR4 to activate systemic inflammation which affects the central nervous system through the extracellular medium (mediated by LPS-binding protein, the cluster of differentiation 14, and myeloid differentiation factor 2 [MD-2]) and intracellularly (TLR4/MyD88/NF-kB and TLR4/TRIF/IRF3 pathways) [64]. The first MyD88-dependent pathway starts from the LPS/MD-2/TLR4 complex located on the plasma membrane, and the second TLR4/TRIF transduction begins in early endosomes after endocytosis of the receptor. The MyD88-dependent pathway is responsible for pro-inflammatory cytokine production; TLR4 recognizes LPS through the accessory molecule MD2, and the intracellular TIR region of TLR4 binds to the carboxyl terminus of MyD88, while the terminus of MyD88 recruits IL-1 receptor-associated kinase-4 (IRAK4), IRAK1, and IRAK2 through homotypic interactions. The activated IRAK4, IRAK1, and IRAK2 are phosphorylated, detach from the MyD88/IRAK complex, and bind to TNF receptor-associated factor 6 (TRAF6). Subsequently, TRAF6 activates the transforming growth factor B-activated kinase (TAK1) complex. Upon activation, TAK1 activates the downstream IκB kinase (IKK), which is made up of two kinases (IKKα and IKKβ), which in turn phosphorylates the NF-κB inhibitor IκBα, leading to NF-κB activation. Finally, free NF-κB is translocated to the nucleus and activates the transcription of inflammatory cytokines such as IL-1, IL-6, and TNF-α [65].

Growing evidence indicates that ASD pathogenesis may involve brain inflammation associated with increased inflammatory biomarkers, such as IL-6 and TNF-α [63, 66, 67]. The protein expression levels of TLR4, phospho-NFκB, p65, and pro-inflammatory cytokines, such as TNFα are dramatically increased in the brain of fetal mice by activating TLR4 signaling in microglia through maternal LPS treatment; these offspring show ASD-like behavior with less social behavior and increased anxiety and repetitive behaviors. However, maternal LPS exposure has no effect on TLR4-knockout mice [64]. The published data support the hypothesis that there may exist a link between TLR-4 activation and NOX-2/ROS upregulation in ASD patients. Activation of TLR-4 by LPS on T-cells *in vitro* leads to enhanced generation of NOX-2-derived ROS *via* the NF-κB pathway, which perhaps plays a pathogenic role under the condition of central nervous system inflammation [68].

Thus, we examined the link in the gut–brain axis to clarify whether gut-derived endotoxins, induced by a “leaky gut”, could be the cause of autism. Our results are in agreement with the findings of the published data [16–19, 45]. LPS levels in plasma were higher in BTBR mice and DSS-treated autistic mice than in metformin-treated BTBR mice. LPS was found in the cell wall of gram-negative bacteria in the gut and high LPS levels reflect an increased intestinal barrier permeability [52]. The expression levels of the pro-inflammatory cytokines MCP-1, IL-1β, and TNF-α and the degree of macrophage infiltration were reduced after metformin treatment. Furthermore, the expression of the upstream regulatory molecules TLR4, MyD88, and NF-κB also decreased. Our previously published articles and other studies also supported this phenomenon [11, 60, 63, 69]. Activated immune cells produce high levels of NF-κB and pro-inflammatory cytokines linked to autism [10]. Children with ASD and BTBR mice have dramatically elevated NF-κB signaling in the brain and periphery [52, 53]. Deng *et al.* reported that metformin alleviates neuroinflammation by decreasing pro-inflammatory cytokines (NF-κB and IL-6) in the hippocampus and rescues the autism-linked behaviors in BTBR mice [69].

**Fig. 10:**
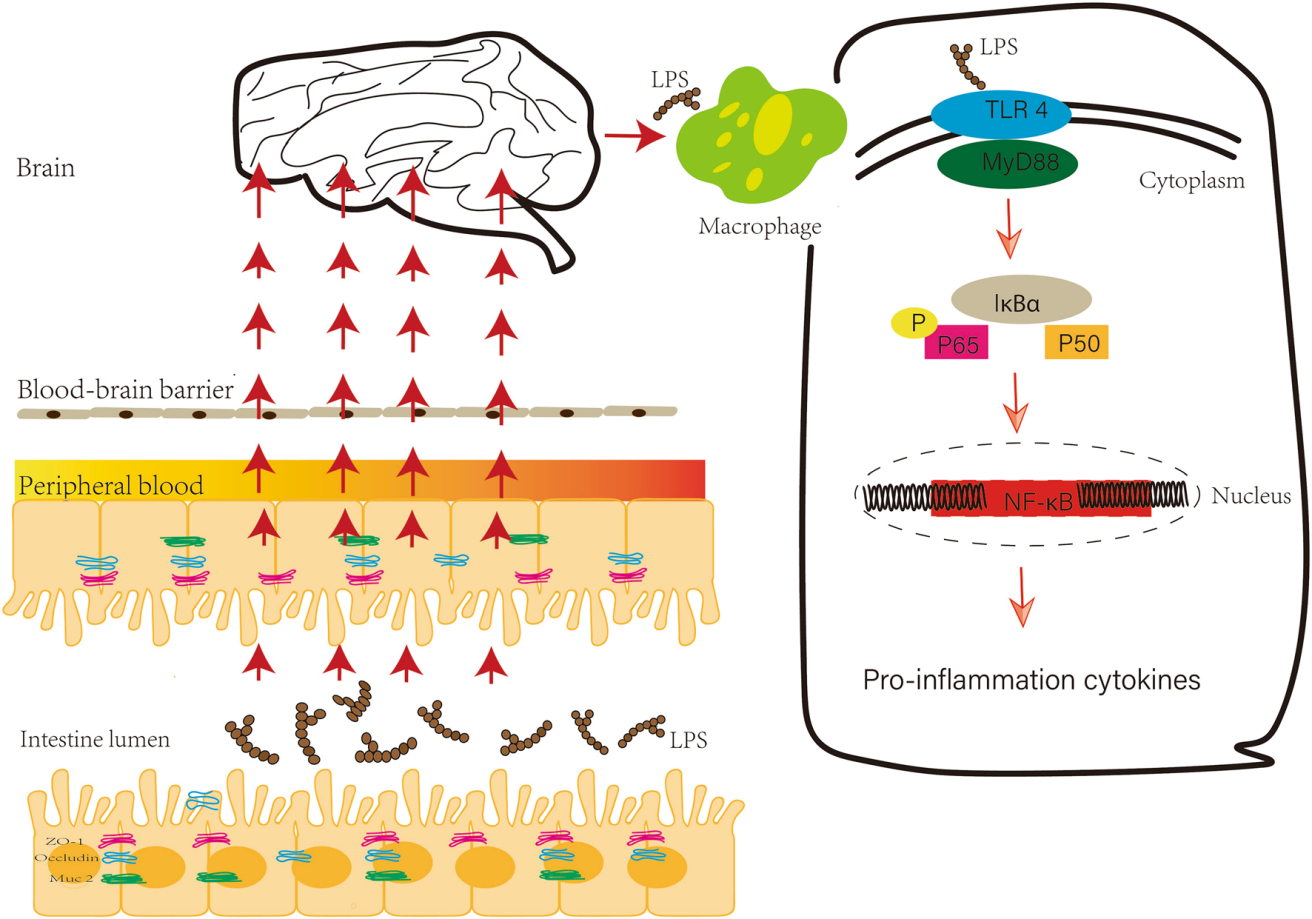
The central pathway of how endotoxin leads to autism spectrum disorder. The leaky gut allows gut endotoxin (LPS) to enter brain tissue. The TLR4/MyD88/NF-κB inflammatory signaling pathway in macrophages promotes brain inflammation and neurodegeneration, contributing to autistic symptoms.

These data supported the hypothesis that increased barrier permeability in autistic mice may transfer LPS to brain tissue to activate the TLR 4/MyD88/NF-κB pathway and further promote the development of the pro-inflammatory environment in the brain leading to neurodegeneration (Fig. 10). Last but not least, the results of our study provided evidence that neuroinflammation may be the main driver behind autism, as already demonstrated by a large number of studies [70–72].

## Supplementary Information

Below is the link to the electronic supplementary material.

Supplementary file1 (PDF 1073 KB)
