# Supplementary material for: Leaky Gut Plays a Critical Role in the Pathophysiology of Autism in Mice by Activating the Lipopolysaccharide-Mediated Toll-Like Receptor 4–Myeloid Differentiation Factor 88–Nuclear Factor Kappa B Signaling Pathway
Source: Neurosci Bull. 2022 Dec 18;39(6):911–28. doi: 10.1007/s12264-022-00993-9 (PMC10264341; doi:10.1007/s12264-022-00993-9)
Supplement: Supplementary file 1 — Supplementary file1 (PDF 1073 KB) [file 12264_2022_993_MOESM1_ESM.pdf]

Supplementary Materials

Table S1 Primers for quantitative PCR

Gene	Forward (5’-3’)	Reverse (5’-3’)
ZO-1[1]	GCCTCATCTCCAGTCCCTTAC	GCAATGGTGGTCCTTCACCT
Occludin[1]	GATGCAGGTCTGCAGGAGTATAA	ATCCTTAATTGGAGTGTTCAGCC
Muc 2[2]	GTGCTGCAATATCACCTCATGT	TGTATGTGATGGAGCCTGAAAC
IL-1β[1]	CTCGTGCTGTCGGACCCAT	CAGGCTTGTGCTCTGCTTGTGA
IL-6[1]	TAGTCCTTCCTACCCCAATTTCC	TTGGTCCTTAGCCACTCCTTC
MCP-1[3]	TCAGCCAGATGCAGTTAACGC	TCTGGACCCATTCCTTCTTGG
TNF-α[1]	CCTGTAGCCCACGTCGTAG	GGGAGTAGACAAGGTACAACCC
β-actin[1]	AGAGGGAAATCGTGCGTGAC	CAATAGTGATGACCTGGCCGT
TLR4[4]	CCTGATGACATTCCTTCT	AGCCACCAGATTCTCTAA
MyD88[4]	GCCAGAGTGGAAAGCAGTGT	TATCGTTGGGGCAGTAGCAG
NF-κB[5]	TCAGGAAGAGGTTTGGATGC	AGCCCCTAATACACGCCTCT

References:

1. Deng J, Chen Y, Zeng L. P054 Metformin protects against intestinal barrier disruption via AMPKα1-dependent inhibition of JNK signaling pathway. J Crohns Colitis 2017, 11: S105.

2. Floyd AM, Zhou X, Evans C, Rompala OJ, Zhu LX, Wang MW, *et al*. Mucin deficiency causes functional and structural changes of the ocular surface. PLoS One 2012, 7: e50704.

3. Yi HS, Eun HS, Lee YS, Jung JY, Park SH, Park KG, *et al*. Treatment with 4-methylpyrazole modulated stellate cells and natural killer cells and ameliorated liver fibrosis in mice. PLoS One 2015, 10: e0127946.

4. Chen L, Sha ML, Li D, Zhu YP, Wang XJ, Jiang CY, *et al*. Relaxin abrogates renal interstitial fibrosis by regulating macrophage polarization via inhibition of Toll-like receptor 4 signaling. Oncotarget 2017, 8: 21044–21053.

5. Deng HD, Kuang P, Cui HM, Luo Q, Liu H, Lu YJ, *et al*. Sodium fluoride induces apoptosis in mouse splenocytes by activating ROS-dependent NF-κB signaling. Oncotarget 2017, 8: 114428–114441.

Table S2 List of antibodies

Antibody	Species	Dilution	Supplier	Catalog Number
ZO-1	Rabbit	1:100	Abcam	ab96587
Occludin	Rabbit	1:100	Abcam	ab224526
Muc 2	Rabbit	1:100	Thermo Fisher	PA5-103083

Supplemental Figures and Figure Legends

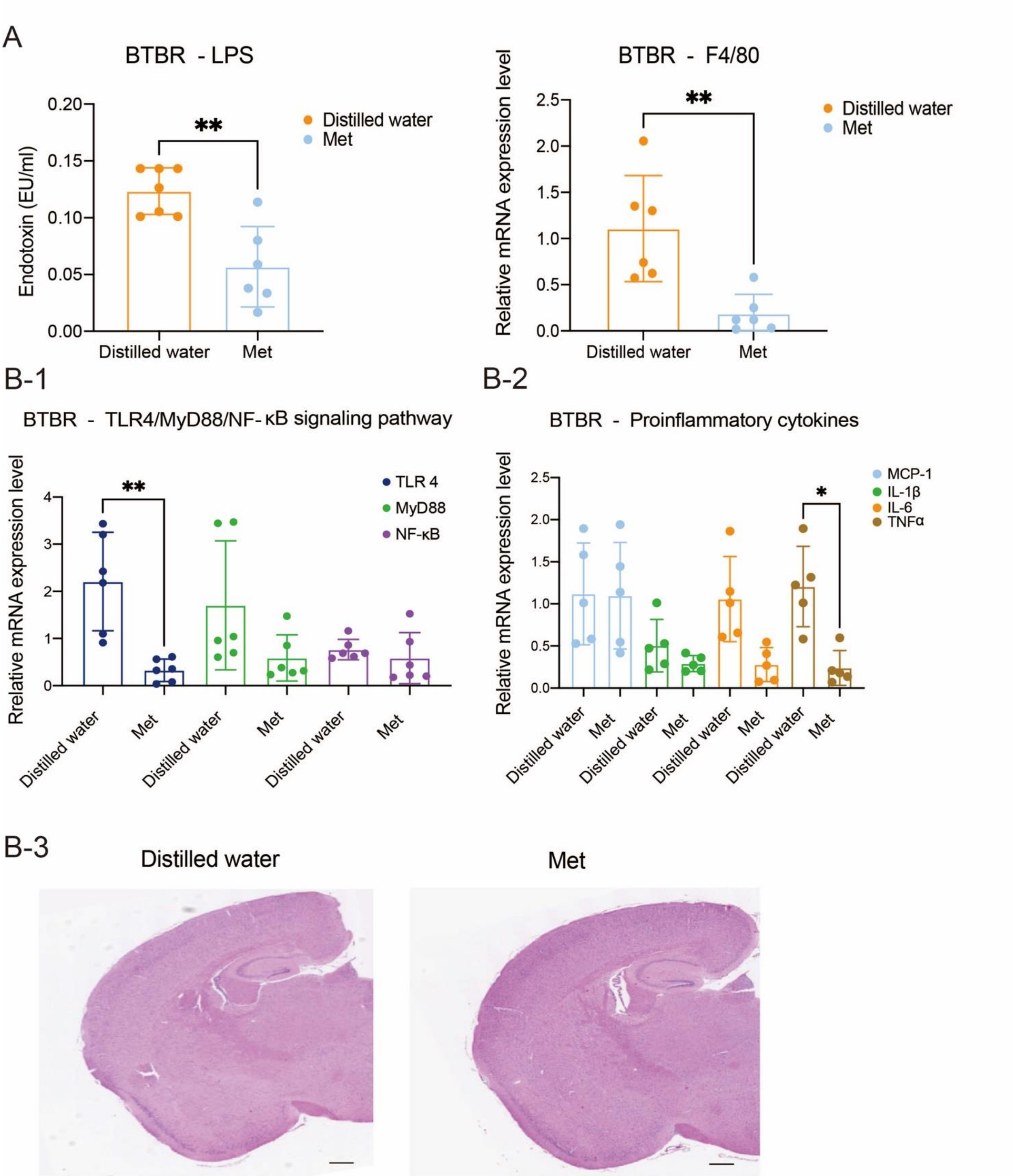

Fig. S1 Effects of metformin administration alone on the intracranial inflammatory environment. Metformin (Met) has no deleterious effect on brain structure and does not raise pro-inflammatory cytokine levels in our autistic animal model (n = 5–6 per treatment). The morphology of brain tissue is not altered in BTBR mice treated with metformin. TNF-α, as well as TLR4, a component in the

upstream pro-inflammatory signaling cascade, are both significantly reduced. Data are shown as the mean ± SEM. *P <0.05, **P <0.01, ***P <0.001, two-tailed unpaired Student's t test. The images were captured by confocal microscopy. Scale bars, 1mm.

A

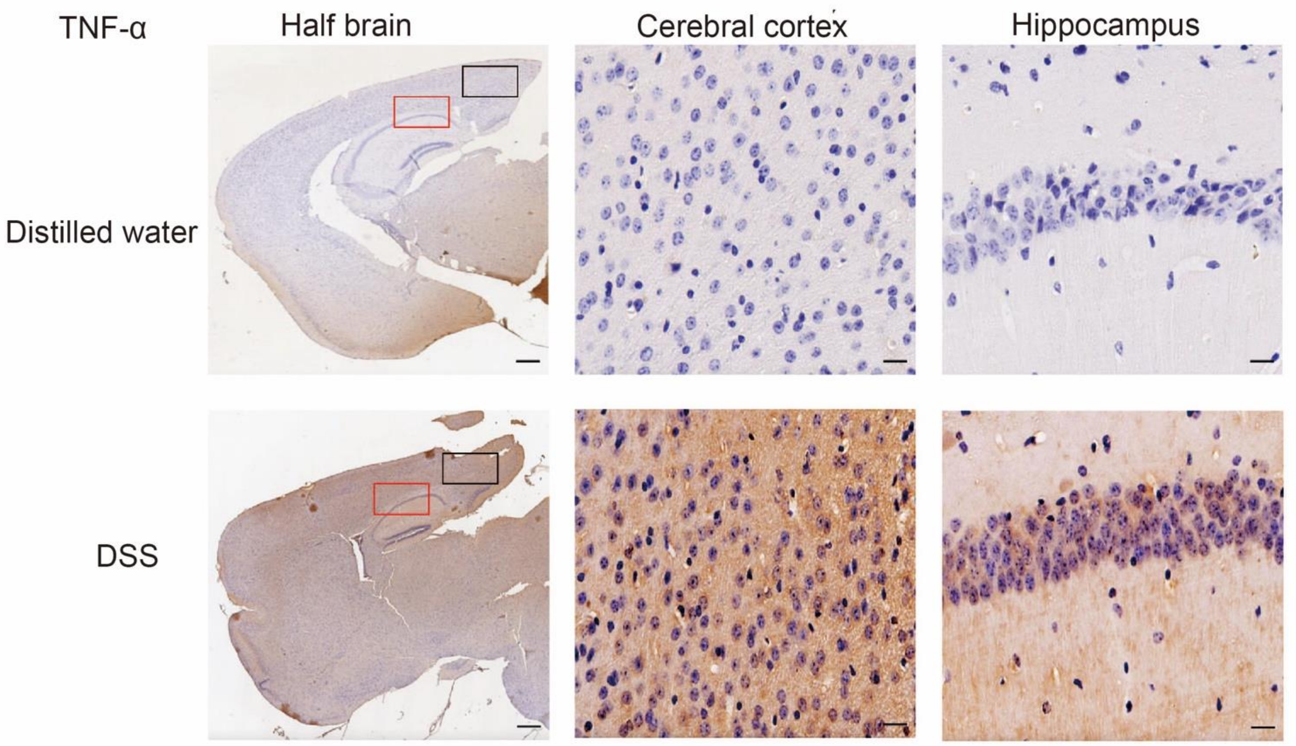

B-1

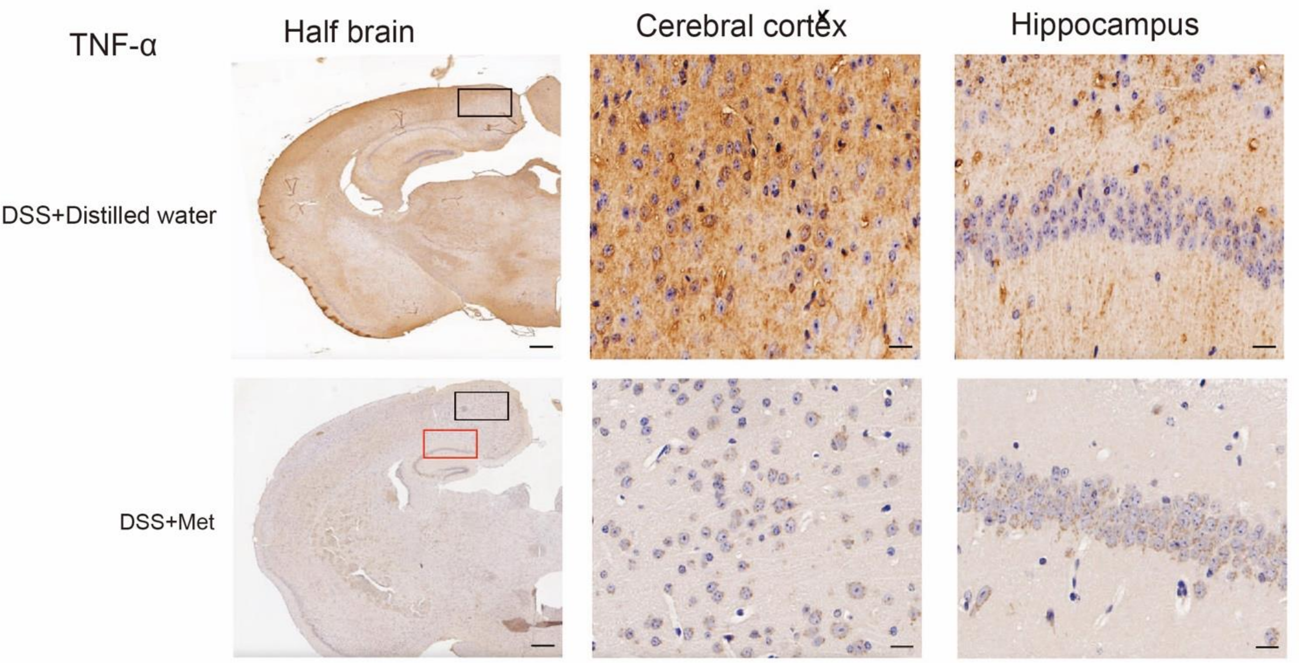

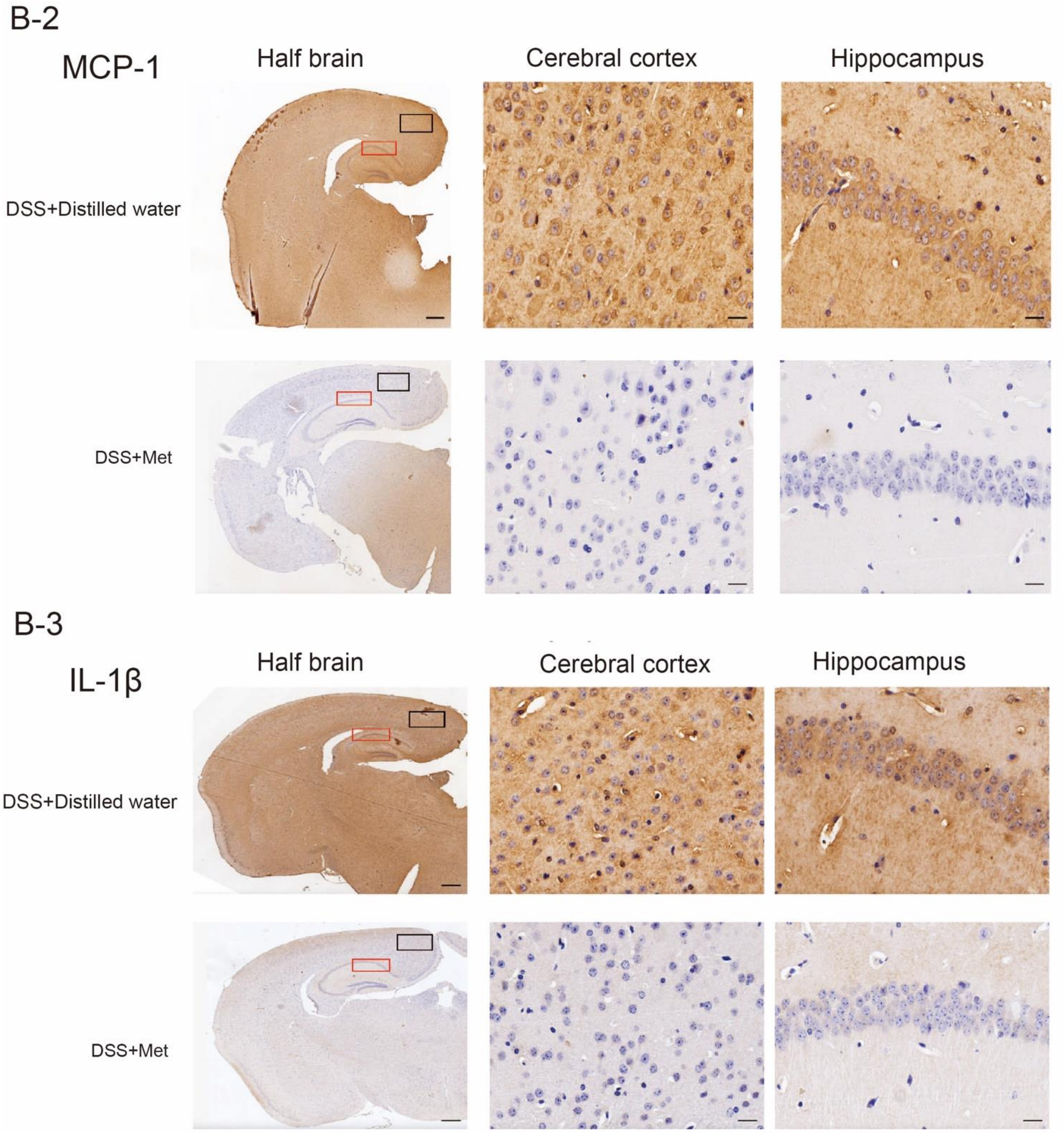

Fig. S2 Effect of intestinal permeability on immunoreactivity in CA1 of the hippocampus and the dorsomedial cerebral cortex (immunohistochemical staining). Brain sections from DSS and Metformin plus DSS groups are immunostained with antibodies against the MCP-1, IL-1β, and TNFα (**A**, **B**) (*n* = 4–5 per treatment). The images were captured by confocal microscopy. Scale bars,1 mm (left graph) and 10 μm (insets for the cerebral cortex and hippocampus separately in the left graph).

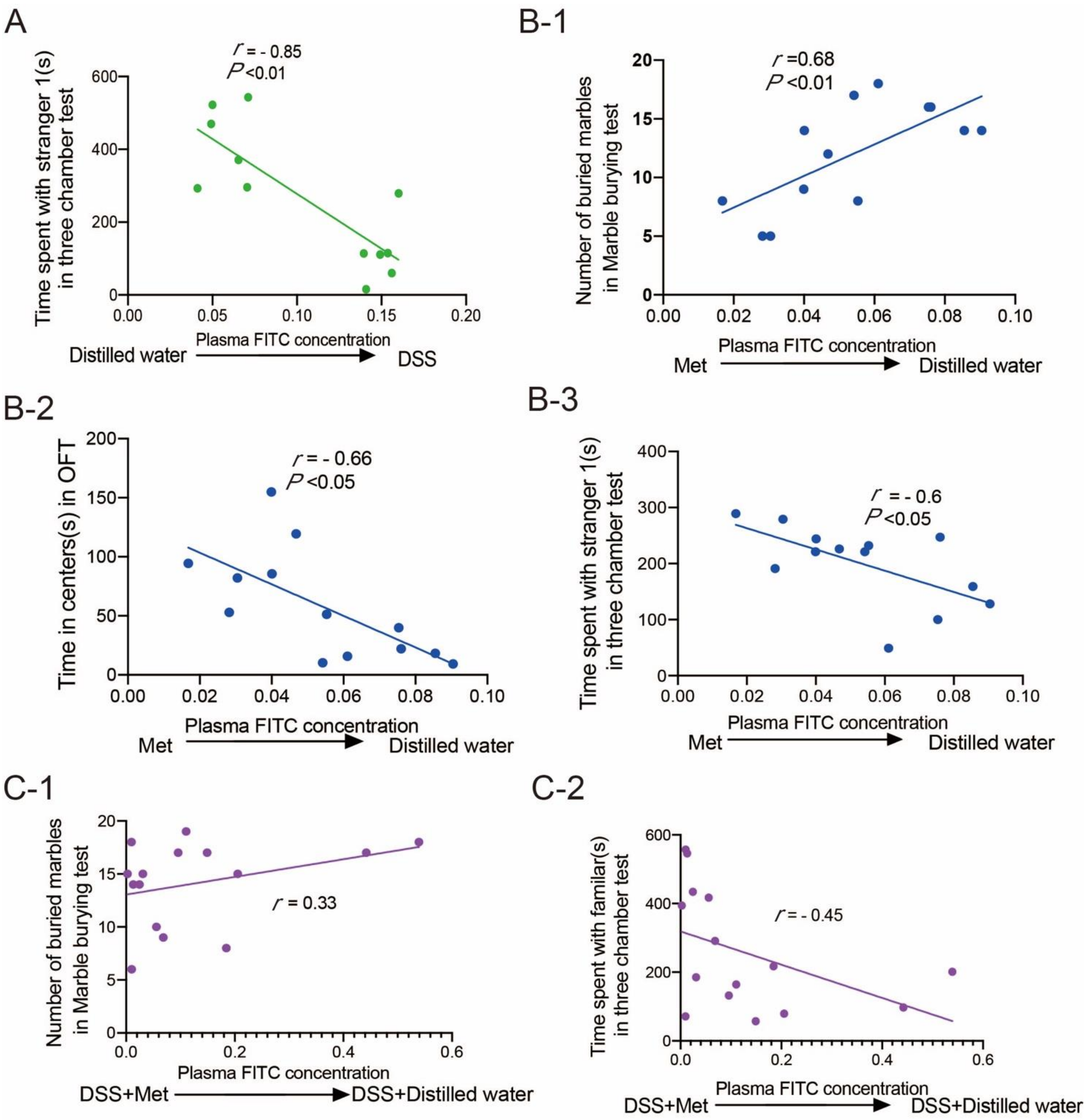

Fig. S3 Correlation analysis of intestinal barrier permeability and autistic behavior (n = 6–8 per treatment).
